# *Vivaxin* genes encode highly immunogenic, non-variant antigens on the *Trypanosoma vivax* cell-surface

**DOI:** 10.1371/journal.pntd.0010791

**Published:** 2022-09-21

**Authors:** Alessandra Romero-Ramirez, Aitor Casas-Sánchez, Delphine Autheman, Craig W. Duffy, Cordelia Brandt, Simon Clare, Katherine Harcourt, Marcos Rogério André, Kayo José Garcia de Almeida Castilho Neto, Marta M. G. Teixeira, Rosangela Zacharias Machado, Janine Coombes, Robin J. Flynn, Gavin J. Wright, Andrew P. Jackson

**Affiliations:** 1 Institute of Infection, Veterinary and Ecological Sciences, University of Liverpool, Liverpool, United Kingdom; 2 Department of Tropical Disease Biology, Liverpool School of Tropical Medicine, Liverpool, United Kingdom; 3 Wellcome Trust Sanger Institute, Wellcome Genome Campus, Hinxton, United Kingdom; 4 Department of Biology, Hull York Medical School, York Biomedical Research Institute, University of York, York, United Kingdom; 5 Department of Pathology, Reproduction and One Health, Faculty of Agrarian and Veterinary Sciences, São Paulo State University (UNESP), Jaboticabal, Sao Paulo, Brazil; 6 Department of Parasitology, Institute of Biomedical Sciences, University of Sao Paulo, Sao Paulo, Sao Paulo, Brazil; 7 School of Pharmacy and Life Sciences, The Robert Gordon University, Aberdeen, United Kingdom; 8 Waterford Institute of Technology, Waterford, Ireland; Hunter College, CUNY, UNITED STATES

## Abstract

*Trypanosoma vivax* is a unicellular hemoparasite, and a principal cause of animal African trypanosomiasis (AAT), a vector-borne and potentially fatal livestock disease across sub-Saharan Africa. Previously, we identified diverse *T*. *vivax*-specific genes that were predicted to encode cell surface proteins. Here, we examine the immune responses of naturally and experimentally infected hosts to these unique parasite antigens, to identify immunogens that could become vaccine candidates. Immunoprofiling of host serum shows that one particular family (Fam34) elicits a consistent IgG antibody response. This gene family, which we now call *Vivaxin*, encodes at least 124 transmembrane glycoproteins that display quite distinct expression profiles and patterns of genetic variation. We focused on one gene (*viv-β8*) that encodes one particularly immunogenic vivaxin protein and which is highly expressed during infections but displays minimal polymorphism across the parasite population. Vaccination of mice with VIVβ8 adjuvanted with Quil-A elicits a strong, balanced immune response and delays parasite proliferation in some animals but, ultimately, it does not prevent disease. Although VIVβ8 is localized across the cell body and flagellar membrane, live immunostaining indicates that VIVβ8 is largely inaccessible to antibody in vivo. However, our phylogenetic analysis shows that vivaxin includes other antigens shown recently to induce immunity against *T*. *vivax*. Thus, the introduction of vivaxin represents an important advance in our understanding of the *T*. *vivax* cell surface. Besides being a source of proven and promising vaccine antigens, the gene family is clearly an important component of the parasite glycocalyx, with potential to influence host-parasite interactions.

## Author summary

Animal African trypanosomiasis (AAT) is an important livestock disease throughout sub-Saharan Africa and beyond. AAT is caused by *Trypanosoma vivax*, among other species, a unicellular parasite that is spread by biting tsetse flies and multiplies in the bloodstream and other tissues, leading to often fatal neurological conditions if untreated. Although concerted drug treatment and vector eradication programmes have succeeded in controlling Human African trypanosomiasis, AAT continues to adversely affect animal health and impede efficient food production and economic development in many less-developed countries. In this study, we attempted to identify parasite surface proteins that stimulated the strongest immune responses in naturally infected animals, as the basis for a vaccine. We describe the discovery of a new, species-specific protein family in *T*. *vivax*, which we call *vivaxin*. We show that one vivaxin protein (VIVβ8) is surface expressed and retards parasite proliferation when used to immunize mice, but does not prevent infection. Nevertheless, we also reveal that vivaxin includes another protein previously shown to induce protective immunity (IFX/VIVβ1). Besides its great potential for novel approaches to AAT control, the vivaxin family is revealed as a significant component of the *T*. *vivax* cell surface and may have important, species-specific roles in host interactions.

## Introduction

African trypanosomes (*Trypanosoma* subgenus *Salivaria*) are unicellular flagellates and obligate hemoparasites. *Trypanosoma vivax* is one of several African trypanosome species that cause animal African trypanosomiasis (AAT), a vector-borne disease of livestock that is endemic across sub-Saharan Africa, as well as found sporadically in South America [1–2]. Cyclical transmission of *T*. *vivax* by tsetse flies (*Glossina* spp.), or mechanical transmission by diverse other biting flies, leads to an acute, blood-borne parasitaemia and subsequent chronic phases during which parasites disseminate to various tissues, the central nervous system in particular [1–5]. AAT is a potentially fatal disease characterised by acute inflammatory anaemia and various reproductive, neural and behavioural syndromes during chronic phase [6–7]. The impact of the disease on livestock productivity, food security and the wider socio-economic development of endemic countries, is profound and measured in billions of dollars annually [8]. Thus, AAT is rightly considered one of the greatest challenges to animal health in these regions [9–10].

Strategies to prevent AAT are typically based around vector control, using insecticides, traps or pasture management, in combination with prophylaxis with trypanocidal drugs [11]. However, widespread drug resistance and the on-going cost of maintaining transnational control means that a vaccine is the preferred, sustainable solution [12–13]. African trypanosome infections are, however, far from an ideal target for vaccination for two reasons. First, antigenic variation of the Variant Surface Glycoprotein (VSG) enveloping the trypanosome cell leads to immune evasion and immunization with VSG fails to protect against heterologous challenge [14]. Second, chronic infection leads to an immunosuppressive environment and ablation of memory B-cells [13].

Successful recombinant vaccines exist for other pathogens that are capable of antigenic switching, for example, hemagglutinin of influenza [15], hepatitis C [16] outer surface antigens of *Borrelia* [17] and the circumsporozoite protein of *Plasmodium falciparum* [18]. These vaccines are based on pathogen surface antigens that elicit dominant immune responses in natural infections. Thus, while antigenic variation of trypanosomes specifically precludes whole-cell vaccine approaches, recombinant vaccines might work if based on non-VSG antigens exposed to the immune system during infections. Yet, most experiments using various conserved and invariant trypanosome proteins [19–21] have not led to robust protective immunity, causing the very plausibility of African trypanosome vaccines to be questioned [22]. Recently, however, in a systematic screen of recombinant subunit vaccines based on *T*. *vivax* non-VSG surface antigens, we identified a *T*. *vivax*-specific, invariant flagellum antigen (IFX) that induced long-lasting protection in a mouse model. This immunity was passively transferred with immune serum, and recombinant monoclonal antibodies to IFX could induce sterile protection [23].

In this study, we continue our evaluation of *T*. *vivax* antigens using a complementary approach, beginning by analysing the naturally occurring antibody responses to *T*. *vivax*-specific surface proteins. We previously categorized genes encoding *T*. *vivax*-specific, cell-surface proteins that were not VSG (‘TvCSP’) into families, named Fam27 to Fam45 inclusive [24]. We showed that many of these TvCSP families (e.g. Fams 29, 30, 32, 34 and 38) are abundant and preferentially expressed in bloodstream-form parasites [25]. Our aim here is to identify candidates for recombinant vaccine development through four objectives: (1) to assay serum antibody from naturally infected animals using a custom TvCSP peptide array; (2) to produce recombinant protein for immunogenic TvCSP using a mammalian expression system; (3) to vaccinate and challenge with *T*. *vivax* in a mouse model; and 4) to examine the cell-surface localisation of TvCSP using immunofluorescent and electron microscopy.

We show that one TvCSP family of 124 paralogous genes encoding putative type-1 transmembrane proteins are especially immunogenic in natural infections, and we name this family *vivaxin*. Vaccination with recombinant vivaxin proteins produces a robust, mixed immune response in mice that significantly reduces parasite burden, but without ultimately preventing infection. We show that at least one vivaxin family member is found on the extracellular face of the plasma membrane of *T*. *vivax* bloodstream-stage trypomastigotes, and therefore, aside from its utility as a vaccine candidate, vivaxin is likely to be an abundant component of the native *T*. *vivax* surface coat, alongside VSG.

## Methods

### Ethics statement

All mouse experiments were performed under UK Home Office governmental regulations (project licence numbers PD3DA8D1F and P98FFE489) and European directive 2010/63/EU. Research was ethically approved by the Sanger Institute Animal Welfare and Ethical Review Board. Mice were maintained under a 12-h light/dark cycle at a temperature of 19–24°C and humidity between 40 and 65%. The mice used in this study were 6–14-week-old male and female *Mus musculus* strain BALB/c, which were obtained from a breeding colony at the Research Support Facility, Wellcome Sanger Institute.

### Design and production of TvCSP peptide microarray

The array design included 63 different *T*. *vivax* Y486 antigens that are not VSG (42 representatives of TvCSP multi-copy families and 21 *T*. *vivax*-specific, single copy genes with predicted cell surface expression). We selected these 63 proteins to ensure that the array included multiple representatives of all putative *T*. *vivax*-specific cell surface gene families, as well as single-copy genes, that were defined in our previous work and strongly expressed in mouse bloodstream infections [24–25]. The microarrays comprised 600 peptides printed in duplicate, each 15 amino acids long with a peptide-peptide overlap of 14 amino acids, and manufactured by PEPperPRINT (Heidelberg, Germany). Each array included peptides cognate to mouse monoclonal anti-FLAG (M2) (DYKDDDDKAS) and mouse monoclonal anti-influenza hemagglutinin HA (YPYDVPDYAG), displayed on the top left and bottom right respectively, which were used as controls (12 spots each control peptide).

### Infected host serum

Blood serum from trypanosusceptible cattle known, or suspected, to be infected with *T*. *vivax* were obtained from Kenya (N = 24), Cameroon (N = 26) and Brazil (N = 6). African samples came from naturally infected animals in endemic disease areas (although not necessarily infected at the time of sampling), while Brazilian serum came from calves experimentally infected with the Brazilian *T*. *vivax* Lins strain [26]. None of the animals had been treated with trypanocidal drugs prior to serum sampling. Samples were screened with the Very Diag diagnostic test (Ceva-Africa; [27]), which confirmed that they were seropositive for *T*. *vivax*. Negative (uninfected) controls were provided by serum from UK cattle (N = 4), seronegative by diagnostic test. A further negative control for cross-reactivity with *T*. *congolense*, (commonly co-incident with *T*. *vivax*), utilised serum from Cameroonian cattle (N = 11) that were seronegative by diagnostic test for *T*. *vivax*, but seropositive for *T*. *congolense*.

### Immunoprofiling assay

Fifteen of the 57 positive *T*. *vivax* samples to be tested in the microarrays were seropositive for *T*. *vivax* only (i.e. unique infection), while 42 were seropositive for both *T*. *vivax* and *T*. *congolense*. Before applying these to the peptide arrays, one array was pre-stained with an anti-bovine IgG goat secondary antibody (H+L) Cy3 (Jackson ImmunoResearch Laboratories) at a dilution 1:4500 in order to obtain the local background values. Slides were analyzed with an Agilent G2565CA Microarray Scanner (Agilent Technologies, USA) using red (670nm) and green (570nm) channels independently with a resolution of 10um. The images obtained were used to quantify raw, background and foreground fluorescence intensity values for each spot in the array using the PEPSlide Analyzer software (Sicasys Software GmbH, Heidelberg, Germany).

### Immunoprofiling analysis

The limma R package from Bioconductor [28] was used to identify the most immunogenic peptides in livestock serum samples by location and across all samples. The data were extracted directly from the Genepix files (.gpr) produced by the PepSlide Analyzer, using only the green channel intensity data. The “normexp” method was selected for background and normalization between arrays was achieved with vsn [29]. A cut-off threshold was defined according to Valentini *et al*. [30] and applied to the raw response intensity (RRI) values. A filtering step was performed removing control peptides (HA and FLAG) from each array and the RRI values from duplicate spots were averaged. After combining all samples from different locations, and both experimental and natural infections, the difference in RRI values in response to infected versus uninfected serum was assessed for each spot using limma to determine statistical significance (p-value < 0.05) and log2 fold-change. Benjamini and Hochberg’s method for the false discovery rate was applied [31].

### Phylogenetic analysis

All full-length vivaxin gene sequences (n = 81) were extracted from the *T*. *vivax* Y486 reference genome sequence. Amino acid sequences were aligned using Clustalx [32] and then back-translated and manually checked using Bioedit [33], producing a 663 nucleotide codon alignment (221 amino acids). Phylogenies were estimated for both codon and amino acid alignments using Maximum likelihood and Bayesian inference. Maximum likelihood trees were estimated using Phyml [34] with automatic model selection by SMS [35], according to the Akaike Information Criterion. The optimal models were GTR+Γ (α = 3.677) and JTT+Γ (α = 5.568) for codon and protein alignments respectively. Topological robustness was measured using an approximate log-likelihood ratio (aLRT) branch test, as well as 100 non-parametric bootstrap replicates. Raxml [36] was also used to estimate bootstrapped maximum likelihood trees, using unpartitioned GTR+FU+Γ (α = 3.134) and LG+Γ (α = 3.847) models for codon and protein alignments respectively. Bayesian phylogenies were estimated from the same alignments using Phylobayes [37], employing four Markov chains in parallel and a CAT model with rate heterogeneity. A single, divergent sequence (TvY486_0024510) was designated as outgroup because it branches close to the mid-point in all analyses.

### Recombinant protein expression

Protein sequences encoding the extracellular domain and lacking their signal peptide, were codon optimized for expression in human cells and made by gene synthesis (GeneartAG, Germany and Twist Bioscience, USA). The sequences were flanked by unique NotI and AscI restriction enzyme sites and cloned into a pTT3-based mammalian expression vector [38] between an N-terminal signal peptide to direct protein secretion and a C-terminal tag that included a protein sequence that could be enzymatically biotinylated by the BirA protein-biotin ligase [39] and a 6-his tag for purification. The ectodomains were expressed as soluble recombinant proteins in HEK293 cells as described [40–41]. To prepare purified proteins for immunisation, between 50 mL and 1.2L (depending on the level at which the protein was expressed) of spent culture media containing the secreted ectodomain was harvested from transfected cells, filtered and purified by Ni2+ immobilised metal ion affinity chromatography using HisTRAP columns using an AKTAPure instrument (Cytivia, UK). Proteins were eluted in 400mM imidazole as described [42] and extensively dialysed into HBS before quantification by spectrophotometry at 280nm. Protein purity was determined by resolving one to two micrograms of purified protein by SDS-PAGE using NuPAGE 4–12% Bis Tris precast gels (ThermoFisher) for 50 minutes at 200V. Where reducing conditions were required, NuPAGE reducing agent and anti-oxidant (Invitrogen) were added to the sample and the running buffer, respectively. The gels were stained with InstantBlue (Expedeon) and imaged using a c600 Ultimate Western System (Azure biosystems). Purified proteins were aliquoted and stored frozen at -20°C until use.

### Vaccine preparation

VIVβ11, VIVβ14, VIVβ20 and VIVβ8 recombinant proteins were combined independently with one of the three adjuvants to analyze the potential different types of immune responses. The vaccine formulation was prepared by combining 20μg purified antigen with either 100μg Alhydrogel adjuvant (Alum) (vac-alu-250; InvivoGen), Montanide W/O/W ISA 201 VG (Sappic) or 15μg saponin Quil-A (vac-quil; InvivoGen), respectively. In a second experiment (see below), the vaccine was formulated with 50μg VIVβ8 and 15μg Quil-A. Control animals were immunized with the adjuvants only using the same concentration as the antigen-vaccinated groups.

### Mouse immunization and challenge with *T*. *vivax*

Our approach to vaccination-challenge experiments has been described previously [23]. Male BALB/c mice were distributed in groups (n = 3) as follows for the immunization: four groups were immunized with Alum in combination with each antigen, (i.e. VIVβ*/A); and four groups with each antigen co-administrated with Montanide ISA 201 VG, (i.e. VIVβ*/M). There was one control group each for VIVβ*/A or VIVβ*/M-vaccinated group. In addition, female mice were randomly distributed in five groups (n = 8), four of them immunized with Quil-A plus each antigen (i.e. VIVβ*/Q) and one as control group immunized with adjuvant only. Mice from all groups were immunized on days 0, 14 and 28 subcutaneously in two injection sites (100μl/injection). Animals from the VIV*/A and VIV*/M groups were euthanized two weeks after the third immunization (day 42), since, by then, it was clear from post-immunization assays that Quil-A provided the preferred, balanced Th1/Th2 response. The VIVβ*/Q rested for 14 days prior to challenge; at day 42, they were infected intraperitoneally with 10^3^ bioluminescent, bloodstream-form *T*. *vivax* parasites (Y486 strain). The parasites were obtained at day 7 post infection (dpi) from previous serial passages in mice. Briefly, 10μl whole blood was collected and diluted 1:50 with PBS+ 5% D-glucose+10% heparin. After challenge, the animals were monitored daily and quantification of *T*. *vivax* infection was measured by bioluminescent *in vivo* imaging. Subsequently, a second challenge was conducted to confirm the results; two groups of mice (n = 15) with equal numbers of each sex were immunized following the same schedule as before with 50μg VIVβ8 + 15μg Quil-A and adjuvant only respectively, prior to challenge on day 74.

### In vivo imaging

Our approach to in vivo imaging has been described previously [23]. Briefly, animals were injected daily starting at 5dpi and 6dpi for the first and second challenge respectively with luciferase substrate D-luciferin (potassium salt, Source BioScience, UK) diluted in sterile PBS for in vivo imaging and data acquisition. Mice were injected intraperitoneally with 200 μl luciferin solution at a dose of 200mg/kg per mouse 10 minutes before data acquisition. Animals were anaesthetized using an oxygen-filled induction chamber with 3% isoflurane and bioluminescence was measured using the in vivo imaging system IVIS (IVIS Spectrum Imaging System, Perkin Elmer). Mice were whole-body imaged in dorsal position and the signal intensity was obtained from luciferase expressed in *T*. *vivax*. The photon emission was captured with charge coupled device (CCD) camera and quantified using Living Image Software (Xenogen Corporation, Almeda, California) and data were expressed as total photon flux (photons/second).

### Serum collection

Blood was collected from the tail vein of each animal at day 0 (pre-immune sera), day 42 (post-immune sera for VIV*/A and VIV*/M treatment groups) and day 50 (post-immune sera for VIV*/Q challenge group). Sera were isolated from blood by centrifuging the samples for 10min x 3,000rpm and the supernatant was stored at -20°C until used for antibody titration. Spleens were aseptically removed from the VIV*/A and VIV*/M groups 42dpi and from VIV*/Q groups at 50dpi. Spleen tissue was used for *in vitro* antigen stimulation in order to quantify cytokine expression.

### In vitro antigen stimulation and cytokine measurement

Splenocytes were isolated by collecting spleens individually in tubes containing 3ml sterile PBS. Single cell suspensions were generated, and red blood cells lysed using ACK lysis buffer. Cell density was adjusted to 5x10^6^ cells/ml per spleen in complete medium and cultured in 48-well flat-bottom tissue culture plates (Starlab, UK) by seeding 200μl/well each suspension in triplicate. Splenocytes were stimulated with 10μg/ml each antigen diluted in complete medium for 72h at 37°C with 70% humidity and 5% CO_2_. Likewise, cells were also incubated with 10μg/ml Concanavalin A (ConA) or complete medium only as positive and negative controls, respectively. Culture supernatants were harvested after 72h and centrifuged at 2000g for 5min at RT to remove remaining cells. The supernatant was collected and used for the quantification of interferon gamma (IFN- γ), tumour necrosis factor (TNF- α), interleukin-10 (IL-10) and interleukin-4 (IL-4) levels by sandwich ELISA kits (ThermoFisher Scientific). The measurement from unstimulated splenocytes (incubated with medium only) was subtracted from the antigen stimulated cultures with each adjuvant treatment.

### IgG-specific antibody response in mice and natural infections

To identify the presence of specific antibodies in mice sera against the antigens, a titration of IgG1 and IgG2a isotypes was performed by indirect ELISA. Briefly, 96-well streptavidin-coated plates were incubated with each antigen for 1h at RT with 1:250 VIVβ11 and 1:50 VIVβ14/Q, VIVβ20/Q and VIVβ8/Q diluted in reagent diluent (PBS pH 7.4, 0.5% BSA), as described previously [43]. The plates were washed three times with PBS-Tween20 0.05% and two-fold serial dilutions of each serum diluted in reagent diluent were performed, added to each well and incubated for 1h at RT. Plates were washed as before and 100μl/well rabbit anti-mouse IgG1 or IgG2a conjugated to HRP (Sigma-Aldrich, Germany) diluted to 1: 50,000 and 1: 25,000, respectively, were added to the plates and incubated as before. After washing, 100μl/well of 3,3’,5,5’-tetramethylbenzidine (TMB, Sigma-Aldrich, Germany) was incubated for 5 minutes at RT in the dark. The reaction was stopped by adding 50μl/well 0.5M HCl and the absorbance was read at 450nm using a Magellan Infinite F50 microplate reader (Tecan, Switzerland).

The isotype profile against each antigen was also analysed in samples from naturally and experimentally infected cattle. The ELISA protocol used was the same as above performing two-fold serial dilutions in samples from experimental and natural infections. Bound IgG1 and IgG2 antibodies were detected by adding 100μl/well sheep anti-bovine IgG1 or IgG2 HRP (Bio-Rad, USA) at 1:5000 and 1:2500 concentration respectively.

### Cellular localization

Cellular localization of VIVβ8 in *T*. *vivax* bloodstream-forms was determined by indirect immunofluorescence. *T*. *vivax* bloodstream-forms were isolated as described previously [23], adjusted to 2.5x10^6^ cells/ml in PBS+20mM glucose, transferred to poly-L-lysine slides for 10min and fixed in 4% formaldehyde for 30min at RT. A polyclonal antibody against recombinant VIVβ8 was raised in rabbits (BioServUK, Sheffield, UK). Briefly, two rabbits were vaccinated by subcutaneous injection, receiving five injections of 0.15mg VIVβ8 antigen diluted in sterile PBS and co-administrated with Freund’s adjuvant every two weeks (0.75mg total immunization). IgG antibodies were purified by affinity chromatography with a protein A column from antisera collected two weeks after the last boost. The final concentration of the rabbit purified antibody was 5mg/ml. Parasite cells were washed with PBS and blocked with blocking buffer (PBS+1% BSA) for 1h at RT. Either pooled anti-VIVβ8 post-immune mouse sera or purified rabbit anti-VIVβ8 IgGs was used as primary antibody (1:1,000 dilution) in blocking buffer and incubated overnight at 4°C. After washing, cells were incubated for 1h at RT with either secondary goat anti-mouse IgG conjugated with Alexa Fluor-555 (Abcam, UK) (1:500 dilution in blocking buffer) or with secondary Alexa Fluor goat anti-rabbit IgG 555 conjugated in blocking solution. Cells were incubated in 500 ng/ml DAPI (Invitrogen, USA), and/or 1:100 mCLING unspecific staining (Synaptic Systems), washed and mounted in Slow Fade diamond antifade mounting oil. Cells were imaged using a LSM-800 confocal laser scanning microscope (Zeiss). Images were processed using Zen 3.1 (Zeiss), ImageJ [44]. 3D renders were generated from z-tacks using ImarisViewer 9.5.1 (Imaris).

### Electron microscopy

Bloodstream-form *T*. *vivax* parasites were obtained from 8 female BALB/c infected mice (>10^8^ parasites/mL) and enriched by centrifugation in 20mM D-glucose PBS, as described previously [23]. Parasites were washed in 0.1M phosphate buffer and fixed in 4% formaldehyde, 0.2% glutaraldehyde in 0.1M phosphate buffer for 1 hour at RT and kept in fixative solution at 4°C.

Fixative was washed out and cells were pelleted and embedded in 3% gelatine and infiltrated in glucose overnight at 4°C. Embedded cells were cut into <1mm cubes and flash frozen ready for cryosectioning with a Leica UC6 ultramicrotome. Cryosections between 60-80nm were picked up using 2% methyl cellulose/2.3M sucrose at a ratio of 1:1 and deposited on formvar/carbon coated nickel grids. Before labelling, gelatine plates were melted at 37°C for 20 minutes with grids in place. Grids were then moved over the following solutions at RT: 20mM glycine in PBS 4 x 1 minute; 10% goat serum in PBS 1 x 10 minutes; 0.1% BSA 2 x 1 minutes; primary rabbit anti-VIVβ8 polyclonal antibody in 0.1% BSA (1:20 dilution) 30 minutes; 0.1% BSA 4 x 2 minutes; secondary goat anti-rabbit IgG 10nm gold-conjugated 0.1% BSA (1:5 dilution) 30 minutes; 0.1% BSA 5 x 2minutes; deionized water 6 x 1 minute. After treatment on ice with 1% aqueous uranyl acetate x 1 minute followed by 1.8% methylcellulose and 0.3% uranyl acetate, the grids were imaged at 100KV on a FEI Tecnai G2 Spirit with Gatan RIO16 digital camera. The proportion of VIVβ8 localised adjacent to the cell surface relative to the cytoplasm was determined by counting gold particles in parasite cells for which the entire plasma membrane was visible in section and distinguishable from neighbouring cells, and stained with at least five particles (N = 51).

### Live immunostaining

Bloodstream-form *T*. *vivax* were isolated from infected blood with three rounds of centrifugation at 2,000xg for 10 minutes at 4°C, and incubated with primary anti-VIVβ8 (purified rabbit polyclonal, 1:200 dilution) in blocking solution (1% BSA) for 30 minutes at either 4°C or RT. Cells were washed in PBS 20mM glucose by centrifugation and incubated with secondary Alexa Fluor goat anti-rabbit IgG 555 conjugated in blocking solution for 30 minutes at either 4°C or RT. After washing as described above, all cells were fixed in 4% formaldehyde for 30 minutes at RT. Cells were then incubated with 500 ng/mL DAPI DNA counterstain, mCLING unspecific staining (1:100 dilution) and/or 5 mg/mL FITC-conjugated ConA for 15 minutes at RT. After washing, cells were mounted in SlowFade diamond mounting oil.

## Results

### Immunoprofiling of naturally infected livestock serum identifies consistent *T*. *vivax*-specific antigens

**Fig 1:**
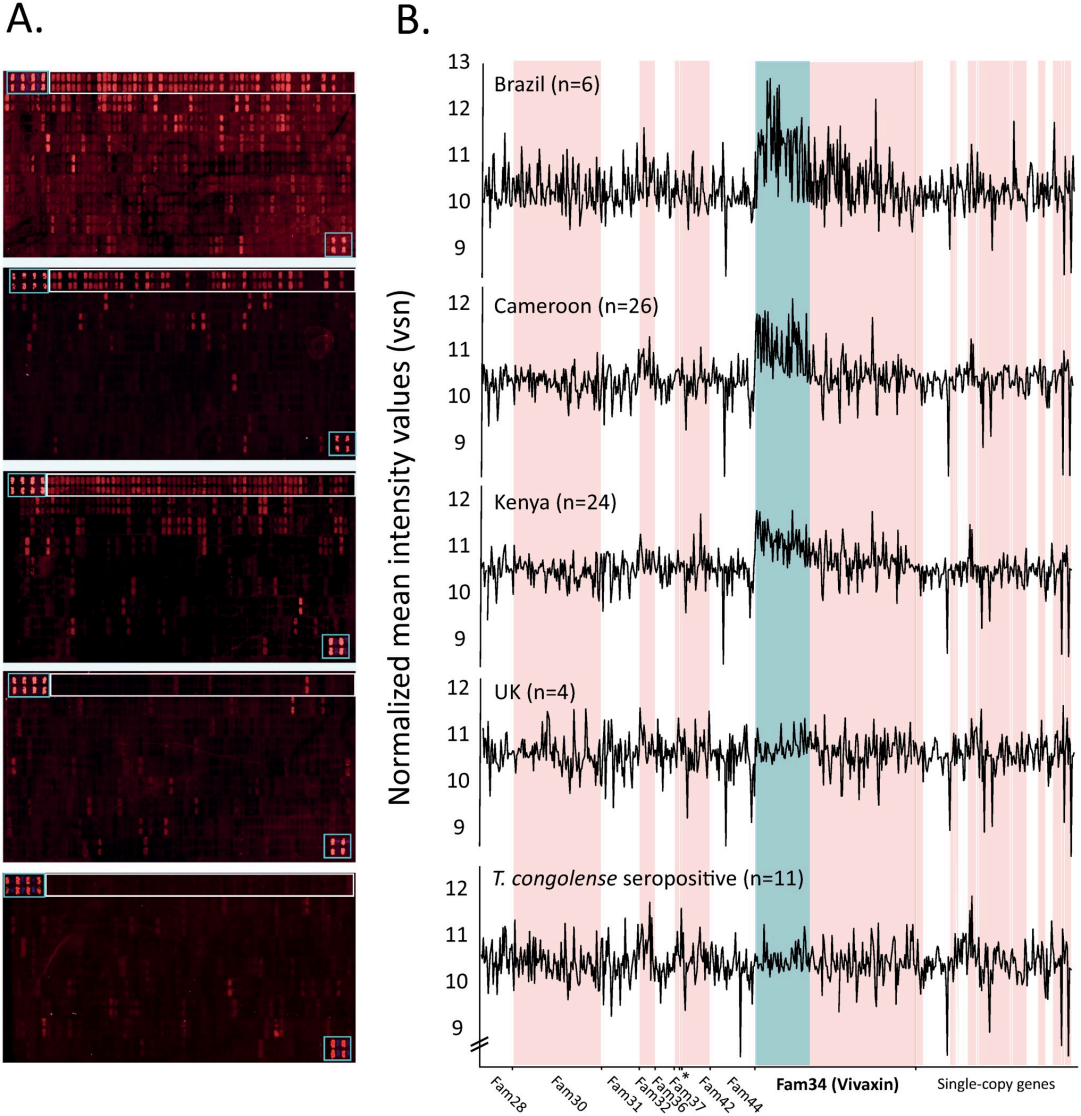
Immuno-profiling of host serum with a *Trypanosoma vivax*-specific antigen array. **(A)** Fluorescence observed after application of host sera to a customised array of 600 peptides representing 63 *T*. *vivax*-specific antigens. From top to bottom, the panels show responses to serum from three locations (Brazil, Cameroon and Kenya respectively), in addition to two negative controls (serum from UK cattle and from Cameroonian cattle that tested *T*. *vivax*-negative but *T*. *congolense*-positive). Control peptides are shown in boxes at top left and bottom right of each array. The top row of the array (boxed) contains peptides exclusively derived from vivaxin proteins. **(B)** Normalized intensity values of immuno-fluorescent responses in (A) are plotted for all array peptides, arranged by gene family. Individual gene families are indicated by alternating white and pink shading. Strongly-responding peptides belonging to vivaxin (Fam34) are indicated by blue shading.

The immuno-reactivity of serum from natural bovine *T*. *vivax* infections in Kenya and Cameroon, as well as experimental bovine infections with Brazilian *T*. *vivax* strains was examined using a custom peptide microarray of 63 putative *T*. *vivax*-specific antigens (S1 Fig). Consistent binding of serum antibodies to peptides in the top two rows of the array was demonstrated for all locations (Fig 1A) with Kenyan, Cameroonian and Brazilian samples displaying a spike in intensity (Fig 1B). The majority of these peptides (51/60) correspond to Fam34 proteins, previously described as a family of putative transmembrane proteins and highly abundant in bloodstream-stage mouse infections [25]. UK cattle that were *T*. *vivax* seronegative and Kenyan cattle that were seropositive for *T*. *congolense* only lacked responses to these peptides (Fig 1).

Table 1 describes the spots with the strongest RRI values (i.e. highest 10%) and shows which spots were significantly greater than fluorescence determined by the negative, uninfected control (see Methods; a complete dataset is provided in S1 Table). Of these 59 strongly responding spots, 45 relate to peptides derived from Fam34 proteins. 29/45 relate to a single family member (hereafter ‘antigen 1’), eight more relate to a second protein (‘antigen-2’), three relate to ‘antigen-3’ and two to ‘antigen-4’. Peptides from antigens 1 and 2 have the highest maximum fold-change in normalized fluorescence intensity relative to the uninfected control, e.g. peptide 46 (4.53) and peptide 35 (2.38) respectively, and four peptides from ‘antigen 1’ have response values that exceed the significance threshold (p < 0.05) after correction for multiple tests (Table 1).

**Table 1:** Parasite peptides displaying the strongest responses to host antibodies in an immuno-profiling assay of *T*. *vivax*-infected serum, across all locations, showing the highest 10% of raw response intensity (RRI) values, ranked by fold change relative to uninfected controls.

Peptide	ID	Family	Antigen	Peptide sequence	RRI	Limma analysis:		Adjusted P
						logFC	P	
46	TvY486_0020520	34	1	DVVVSEESDSELIDL	3045.4	4.53	0.011	0.403
**5**	**TvY486_0020520**	**34**	**1**	**EESDSELIDLAVEAS**	**3563.5**	**3.78**	**0.000**	**0.003**
**9**	**TvY486_0020520**	**34**	**1**	**SEESDSELIDLAVEA**	**3010.5**	**3.30**	**0.000**	**0.039**
3	TvY486_0020520	34	1	VVSEESDSELIDLAV	3717.7	2.46	0.041	0.685
35	TvY486_0003690	34	2	TADDIDAELIEAVTG	3620.2	2.38	0.001	0.117
**23**	**TvY486_0020520**	**34**	**1**	**VLKTGDGGDVVVSEE**	**4676.8**	**2.09**	**0.000**	**0.040**
**11**	**TvY486_0020520**	**34**	**1**	**KTGDGGDVVVSEESD**	**5093.4**	**2.06**	**0.000**	**0.039**
21	TvY486_0020520	34	1	EAVLKTGDGGDVVVS	4006.3	2.00	0.007	0.383
42	TvY486_0003690	34	2	ADDIDAELIEAVTGP	3350.6	1.99	0.056	0.737
28	TvY486_0020520	34	1	SDSELIDLAVEASGQ	2568.7	1.93	0.048	0.709
33	TvY486_0003690	34	2	DDIDAELIEAVTGPA	2674.3	1.89	0.067	0.749
39	TvY486_0003690	34	2	ITADDIDAELIEAVT	4732.8	1.72	0.080	0.765
14	TvY486_0020520	34	1	LKTGDGGDVVVSEES	5596.6	1.66	0.002	0.120
18	TvY486_0020520	34	1	AVLKTGDGGDVVVSE	4245.0	1.46	0.016	0.506
41	TvY486_0003690	34	2	DIDAELIEAVTGPAS	1864.0	1.42	0.036	0.685
15	TvY486_0900440	34	4	VESEDLIDLATQVSE	1824.8	1.38	0.060	0.737
44	TvY486_0039530	34		IHVDGSDLELIELAL	1956.4	1.36	0.074	0.750
108	TvY486_0003690	34	2	VVDITADDIDAELIE	3641.2	1.26	0.057	0.737
80	TvY486_0020520	34	1	GDVVVSEESDSELID	3599.9	1.08	0.142	0.828
205	TvY486_0003690	34	2	KGTADGVQSESGSKT	3075.9	0.97	0.189	0.834
12	TvY486_0020520	34	1	PEAVLKTGDGGDVVV	3607.2	0.85	0.172	0.834
52	TvY486_0020520	34	1	VEKIQSKIKQEGGSA	1860.4	0.81	0.120	0.828
37	TvY486_0020520	34	1	DSELIDLAVEASGQH	1894.9	0.72	0.242	0.901
16	TvY486_0020520	34	1	APRSSADAPLEPTAR	1742.3	0.72	0.170	0.834
25	TvY486_0020520	34	1	PRSSADAPLEPTARD	2097.5	0.64	0.146	0.828
26	TvY486_0020520	34	1	EGGSAPRSSADAPLE	1955.5	0.62	0.251	0.908
97	TvY486_0020520	34	1	KIQSKIKQEGGSAPR	1777.8	0.60	0.176	0.834
38	TvY486_0020520	34	1	ANKPEAVLKTGDGGD	1829.9	0.55	0.298	0.936
61	TvY486_0020520	34	1	KQEGGSAPRSSADAP	1993.4	0.50	0.423	0.977
2	TvY486_0020520	34	1	ESDSELIDLAVEASG	3263.7	0.38	0.614	0.977
32	TvY486_0020520	34	1	NKPEAVLKTGDGGDV	1965.4	0.33	0.515	0.977
4	TvY486_0020520	34	1	GGSAPRSSADAPLEP	1736.6	0.32	0.616	0.977
13	TvY486_0020520	34	1	KPEAVLKTGDGGDVV	2527.5	0.32	0.555	0.977
106	TvY486_0020520	34	1	QSKIKQEGGSAPRSS	1683.9	0.27	0.530	0.977
90	TvY486_0020520	34	1	SKIKQEGGSAPRSSA	1768.0	0.20	0.691	0.977
163	TvY486_0037990	34	3	VEAGEDLMDLVDAVG	1898.7	0.17	0.706	0.977
583	TvY486_0900440	34	4	KGDGEAEKTQAEGKS	2511.5	0.15	0.752	0.977
112	TvY486_0037990	34	3	GVEAGEDLMDLVDAV	2832.7	-0.01	0.992	0.996
85	TvY486_0003690	34	2	VDITADDIDAELIEA	2992.7	-0.01	0.992	0.996
358	TvY486_0037990	34	3	GGVVGVEAGEDLMDL	1950.0	-0.06	0.896	0.994
51	TvY486_0020520	34	1	QEGGSAPRSSADAPL	2219.3	-0.10	0.918	0.994
212	TvY486_0043780	32		KGVNGTETRAGEEVR	2264.3	-0.12	0.736	0.977
48	TvY486_0020520	34	1	GGDVVVSEESDSELI	5227.7	-0.13	0.875	0.994
183	TvY486_0039510	SCG		KDLSPEEVGAYTVFA	3254.6	-0.19	0.754	0.977
288	TvY486_0001040	42		VEEVLRSVEVILESP	1912.6	-0.26	0.670	0.977
599	TvY486_0040160	28		TAVPDDCQVGNDTNS	2134.9	-0.33	0.747	0.977
436	TvY486_0025570	32		WSWYGEMGSFGIFDV	1993.1	-0.44	0.165	0.834
489	TvY486_0042890	32		GWRDQVEYIGDLFSV	1919.9	-0.45	0.260	0.908
572	TvY486_0023840	30		TEWQYDLLRDKIDRI	2203.9	-0.45	0.211	0.859
396	TvY486_0001040	42		LYSLLEVSRVGEEVS	2308.8	-0.53	0.261	0.908
201	TvY486_0039530	34		GSDLELIELALEESP	4407.8	-0.62	0.332	0.966
47	TvY486_0025790	SCG		KDFKEMFIKCSKGDG	2505.6	-0.67	0.147	0.828
192	TvY486_0043780	32		LKGVNGTETRAGEEV	1733.3	-0.79	0.101	0.794
377	TvY486_0016160	SCG		GIDTYVEGLGEIDTL	3511.6	-0.81	0.137	0.828
346	TvY486_0016160	SCG		IGEGIDTYVEGLGEI	2860.3	-0.95	0.040	0.685
455	TvY486_0001040	42		TLYSLLEVSRVGEEV	1742.1	-0.97	0.057	0.737
482	TvY486_0040160	28		VVDKCDPLYQQFLDV	1719.2	-1.11	0.359	0.977
6	TvY486_0020520	34	1	VSEESDSELIDLAVE	3563.1	-1.83	0.351	0.977
427	TvY486_0031450	30		KYDALSTKIGEITIS	2058.8	-2.55	0.008	0.403

Note: RRI is raw response intensity. Significance of response is expressed as log_2_ fold-change relative to fluorescent intensity in uninfected controls. P_adj_ denotes significance after correction for multiple tests (Benjamini). Significant responses shown in bold. SCG: single-copy genes, i.e. *T*. *vivax*-specific genes with predicted cell surface expression that are not members of multi-copy gene families.

Thus, we may conclude that Fam34 is principally responsible for the peak clearly visible in Fig 1B and, in particular, ‘antigen-1’ and ‘antigen-2’ are responsible for 63% of the strongest responses. It may be that our approach has underestimated the immunogenicity of other protein families since the peptide array lacked any post-translational modifications that are common on *T*. *vivax* surface proteins and could contribute to antibody binding. Nevertheless, given the pre-eminence of Fam34 proteins as consistent and robust antigens in natural infections, we focused our search for vaccine targets on this gene family, which we now rename *vivaxin*.

### Vivaxin is a species-specific gene family encoding type-1 transmembrane proteins that do not display antigenic variation

Analysis of vivaxin amino acid sequences with BLAST returns no matches besides *T*. *vivax* itself, and more sensitive comparison of protein secondary structural similarity using HMMER also fails to detect homologs beyond *T*. *vivax*; this confirms that the family is species-specific. Comparison with the *T*. *vivax* Y486 reference genome (TritrypDB release 46) using BLASTp returns 50 gene sequences, while a further 74 homologs are detected by HMMER, which means that vivaxin is the largest *T*. *vivax* cell-surface gene family after VSG [24,45]. These gene sequences range from 1050–1900 bp in length when complete; 43/124 sequences are curtailed by sequence gaps in the current assembly. Only six sequences are predicted to contain internal stop codons, suggesting that pseudogenes are rare. We observe that almost all BLAST matches relate to sub-telomeric loci, (i.e., outside of regular core polycistrons). Previously, in silico predictions based on amino acid sequences indicated that all Fam34 genes encode a type-1 transmembrane protein with a predicted signal peptide and a single hydrophobic domain 15 amino acids from the C-terminus, orientated such that the protein is largely extracellular [24]. We carried out further analysis of antigens 1–4 with PredictProtein [46] and ModPred [47], shown in S2 Fig, that confirm this topology and suggest that the extracellular portion of vivaxin is both N- and O-glycosylated at multiple sites.

**Fig 2:**
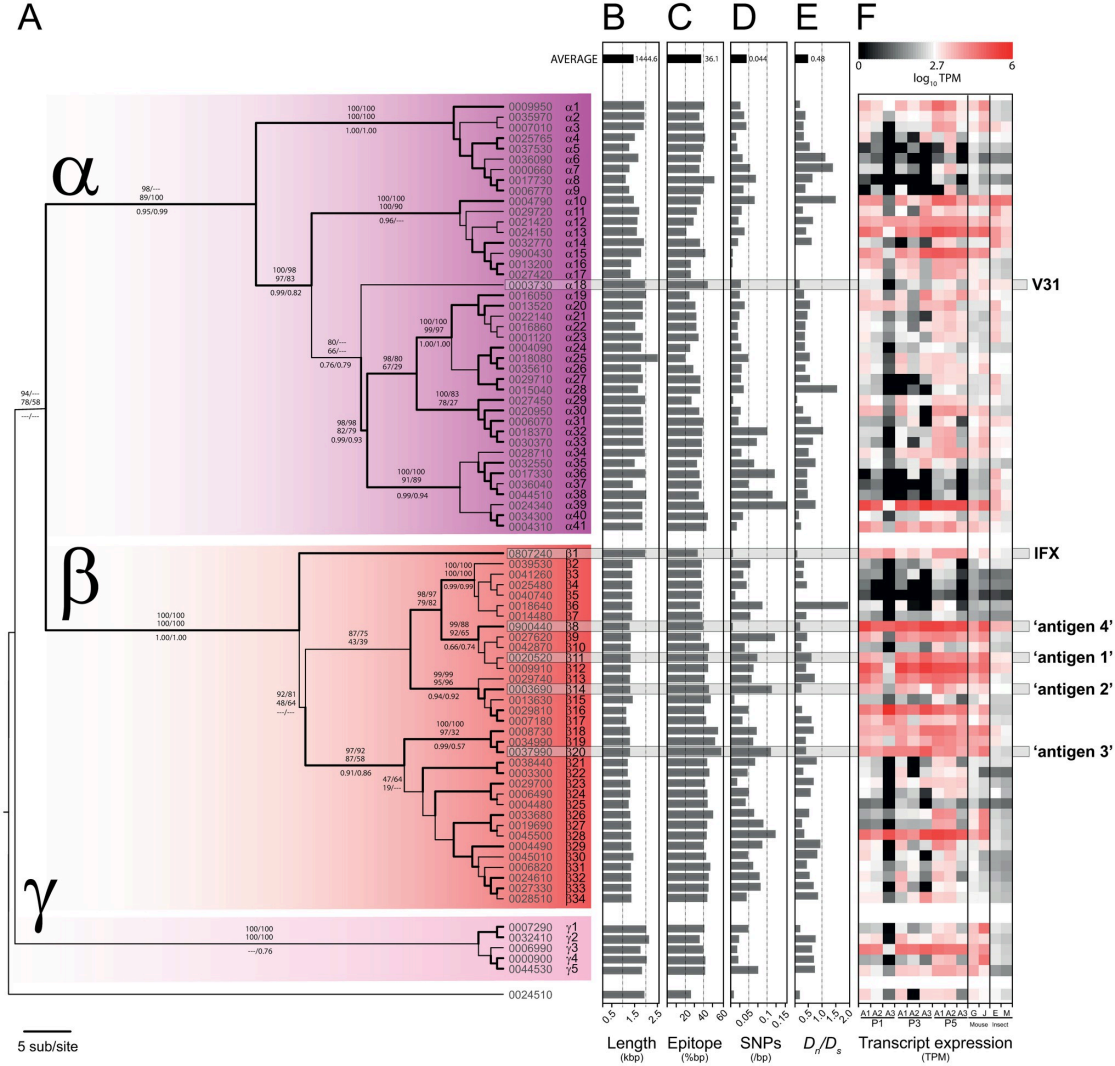
Vivaxin gene family phylogeny and molecular evolution. **(A)** Maximum likelihood phylogeny of vivaxin genes (n = 81) in the *T*. *vivax* Y486 reference genome estimated with a GTR + Γ substitution model (α = 3.677), and divided into three principal sub-families, labelled α, β and γ. The tree is rooted with a divergent sequence (TvY486_0024510) that approximates to the mid-point. Topological robustness is measured by the approximate log-likelihood ratio (aLRT), and indicated by branch thickness. Thick branches subtend nodes with aLRT values > 0.9. Robustness measures are given for major internal nodes: maximum likelihood bootstrap values (> 75) for nucleotide/protein alignments (upper, above branches), neighbour-joining bootstrap values (> 75) for nucleotide/protein alignments (lower, above branches), and Bayesian posterior probabilities (> 0.5) for nucleotide/protein alignments (below branch). At the left of each terminal node are the existing TritrypDB gene identifier (i.e. TvY486_XXXXXXX) and new gene names; the positions of IFX and V31 antigens [23] and four expressed antigens in this study (1–4) are highlighted within horizontal grey boxes. **(B)** Gene length mapped to tree topology. **(C)** Total length of predicted human B-cell epitopes as a proportion of gene length, as inferred by Bepipred linear prediction 2.0 [50]. **(D)** Single nucleotide polymorphisms (SNP) across a panel of 25 *T*. *vivax* strain genomes (as described previously in [48]), as a proportion of gene length. **(E)** Ratio of non-synonymous to synonymous substitutions (*D*_*n*_*/D*_*s*_) inferred from published SNP data [48]. **(F)** Heat maps showing vivaxin gene expression profiles from published *T*. *vivax* transcriptomes [25,48]. The first nine columns show relative transcript abundance during an experimental infection in goats. Three peaks in parasitaemia are shown (first, third and fifth respectively; see [48]), with three replicates for each (A1-A3). Columns 10 and 11 show relative transcript abundance in bloodstream-stage infections in mice using different *T*. *vivax* strains, LIEM-176 [49] and IL1392 [25], respectively. Columns 12 and 13 show transcript abundance in batch transcriptomes of in vitro cultured *T*. *vivax* insect-stages, i.e. epimastigotes (E) and metacyclic-forms (M) respectively [25].

We estimated a Maximum Likelihood phylogeny for 81 full-length vivaxin genes from an alignment of a 221-amino acid conserved region (see Methods). Fig 2A shows that vivaxin sequences group into three robust clades, which we term the α (41 genes), β (34 genes) and γ (5 genes) subfamilies. The genes encoding antigens 1–4 are noted, and are known as *viv-β11*, *-β14*, *-β20* and *-β8* respectively hereafter. The subfamilies consistently differ in length due to the N-terminal (extracellular) domain of vivaxin-α proteins being ~200 amino acids longer than vivaxin-β (Fig 2B). For each gene, the proportion of its protein sequence predicted in silico to be a human B-cell epitope is shown in Fig 2C; on average, 36.1% of a vivaxin protein sequence is predicted to be immunogenic, rising to almost 60% in some cases.

SNPs were identified for each gene using GATK based on variations among 25 clinical *T*. *vivax* genome sequences we published previously ([48]; Fig 2D). These show that, far from being uniformly polymorphic, the population history of vivaxin genes is extremely variable, with some loci being well conserved, indeed almost invariant, across populations. Note that *viv-β11*, *-β14*, *-β20* and *-β8*, (encoding antigens 1–4 respectively), are all among the least polymorphic paralogs. Most loci are predicted to be under purifying selection, and for some including *viv-α15*, *-α29*, as well as the gene encoding antigen-4 (*viv-β8*), this is stringent (i.e. *d*_*n*_*/d*_*s*_ ≈ 0; Fig 2E). The *d*_*n*_*/d*_*s*_ ratio only exceeds 1 for five genes and in only one case (*viv-α10*) does the positively-selected gene show evidence for expression. Fig 2 indicates that, first, there is consistent variation across the family in gene function, with some loci being essential while others are less so, and second, that this variation has been stable throughout the species history, and not subject to assortment or homogenisation by recombination.

### Vivaxin loci display conserved variation in gene expression profiles

We examined RNAseq data from multiple previous experiments to consider functional variation among vivaxin genes. Fig 2F shows transcript abundance at sequential points of an experimental goat infection ([48]; first nine columns), also in two separate experimental infections in mice ([25, 49]; columns 10 and 11) and, finally, in epimastigote (E) and metacyclic (M) parasite stages ([25]; columns 12 and 13 respectively). Most vivaxin genes are expressed weakly in fly stages, confirming that this is predominantly a bloodstream-stage family; although there are exceptions (see *viv-α36* and α*38*). Some genes are expressed rarely in all situations, such as *viv-α6*, -*α8*, and -*β3–7*, indicating that they may be non-functional (in the case of *viv-α6* and -*α8*, these genes do indeed have internal stop codons). Conversely, genes such as *viv-α10*, *α12*, *α39* and *β11–12* are expressed constitutively. These genes remain abundant across sequential peaks of bloodstream infections, and across life stages, and indeed, across experiments using different parasite strains and hosts. This clearly indicates that, like many multi-copy surface antigen gene families, expression levels vary markedly among vivaxin genes, but, unlike other gene families, these differences are not dynamic. There is a cohort of vivaxin loci that are routinely active and orders of magnitude more abundant than most other paralogs. Since many vivaxin genes are expressed simultaneously, this indicates that they are not variant antigens with monoallelic expression such as VSG; moreover, the presence of specific genes that are seemingly constitutively expressed in different infections and parasite strains, with minimal polymorphism, shows that some vivaxin proteins are not variant antigens at all.

Most interestingly, Fig 2F shows that transcripts of *viv-β11*, *-β14*, *-β20* and *-β8* are among the most abundant vivaxin transcripts in all conditions, perhaps explaining why they elicit some of the strongest immune responses. In particular, *viv-β8* transcripts, which encode antigen-4, are perhaps the most abundant, often two orders of magnitude more abundant than most other loci. Taking the results together, we see that the most immunogenic Fam34 proteins are also among the most abundant vivaxin transcripts and among the most evolutionarily conserved vivaxin genes. Hence, our decision to focus on antigens 1–4 as potential subunit vaccines was based on the balance of immunoprofiling, gene expression and polymorphism data.

### Recombinant expression of four β-vivaxin proteins

To determine whether the vivaxin family could elicit protective immune responses in the context of a subunit vaccine and murine model of *T*. *vivax* infection we first expressed the entire ectodomains of four vivaxin proteins using a mammalian expression system. The four recombinant soluble *T*. *vivax* proteins were purified using their C-terminal 6-histidine tags from spent tissue culture supernatants, quantified and resolved by SDS-PAGE to check their mass and integrity (S3 Fig). As expected, each protein preparation resolved as a mixture of different glycoforms between 50 and 55kDa which agreed well with their predicted molecular mass. Together, these data show we were able to express and purify recombinant vivaxin proteins corresponding to the entire ectodomain using a mammalian expression system.

### Immunization with VIVβ8 produces a balanced antibody response

Having expressed recombinant vivaxin proteins, we examined their potential for vaccination. Initially, to establish a robust seroconversion, we inoculated BALB/c mice with our four recombinant vivaxin proteins in combination with multiple adjuvants and measured serum IgG1 and IgG2a antibody titres by indirect ELISA. Independently of adjuvant or antigen, antibody titre increased upon booster immunization indicating these antigens were immunogenic in mouse.

Antibody titres showed a significant increase (p < 0.001) in both IgG1 and IgG2a-specific antibody compared with the pre-immune for all antigens regardless the adjuvant used (S4 Fig). However, adjuvant choice has a significant effect on antibody titres. Quil-A produced significantly higher IgG2a titres than either Montanide or Alum when applied with all antigens. Overall, mice vaccinated with Alum and Montanide elicited higher titres of IgG1 than IgG2a (ratios = 2.11 and 1.58 respectively) suggesting a Th2-biased immune response, while Quil-A came closest to producing an equal ratio of isotype titres (ratio = 1.03), which indicates a mixed Th1/2-type response.

To compare the antibody responses to immunization with those observed for natural and experimental infections, we measured IgG1 and IgG2a titres in livestock serum seropositive for *T*. *vivax* (see above). Naturally-infected cattle from Cameroon and Kenya displayed significantly higher IgG1-specific titres than seronegative UK cattle (p < 0.05) for all four vivaxin antigens (S5 Fig). Experimentally infected cattle from Brazil showed a similar pattern to natural infections with a higher IgG1 than IgG2 antibody levels. Conversely, in most cases, anti-IgG2a responses for each antigen were not significantly greater than the negative control. These results indicate that, while vivaxin is strongly immunogenic in natural infections, it elicits a largely Th2-type response, similar to that produced by immunization with Montanide or alum, but that immunization with Quil-A using any of the recombinant vivaxin antigens can produce a more balanced effect.

Cytokine expression provides further evidence for the type of immune response elicited by immunization. The concentrations of four cytokines (TNF-α, IFN-γ, IL-10 and IL-4) were measured in ex vivo mouse splenocyte cultures after stimulation with each vivaxin antigen, co-administrated with one of three adjuvants. All cytokines were undetectable in splenocytes cultured in media only, but after re-stimulation with an antigen, cytokine concentration increased significantly (p < 0.0001; S6 Fig). Immunization with each antigen, regardless of adjuvant, produced high TNF-α concentration, with no significant differences between antigens (p > 0.05). IFN-γ concentration was greater in all animals immunized with Quil-A, which produced a similar response to the positive control group stimulated with ConA. The expression of IL-10 was also dependent on the adjuvant; it was significantly greater when antigens were co-administered with Quil-A (p < 0.001 and p < 0.0001 for all cases). IL-4 displayed the lowest expression levels of all; the only appreciable difference being for VIVβ20 co-administered with Quil-A compared to all other antigens (p < 0.001 for all cases). These results further indicate that immunization with vivaxin combined with Quil-A produces hallmarks of a Th1 response, which has been observed to be necessary for controlling trypanosome infections [51–53].

### Vaccination with VIVβ8 delays parasite proliferation but does not prevent infection

**Fig 3:**
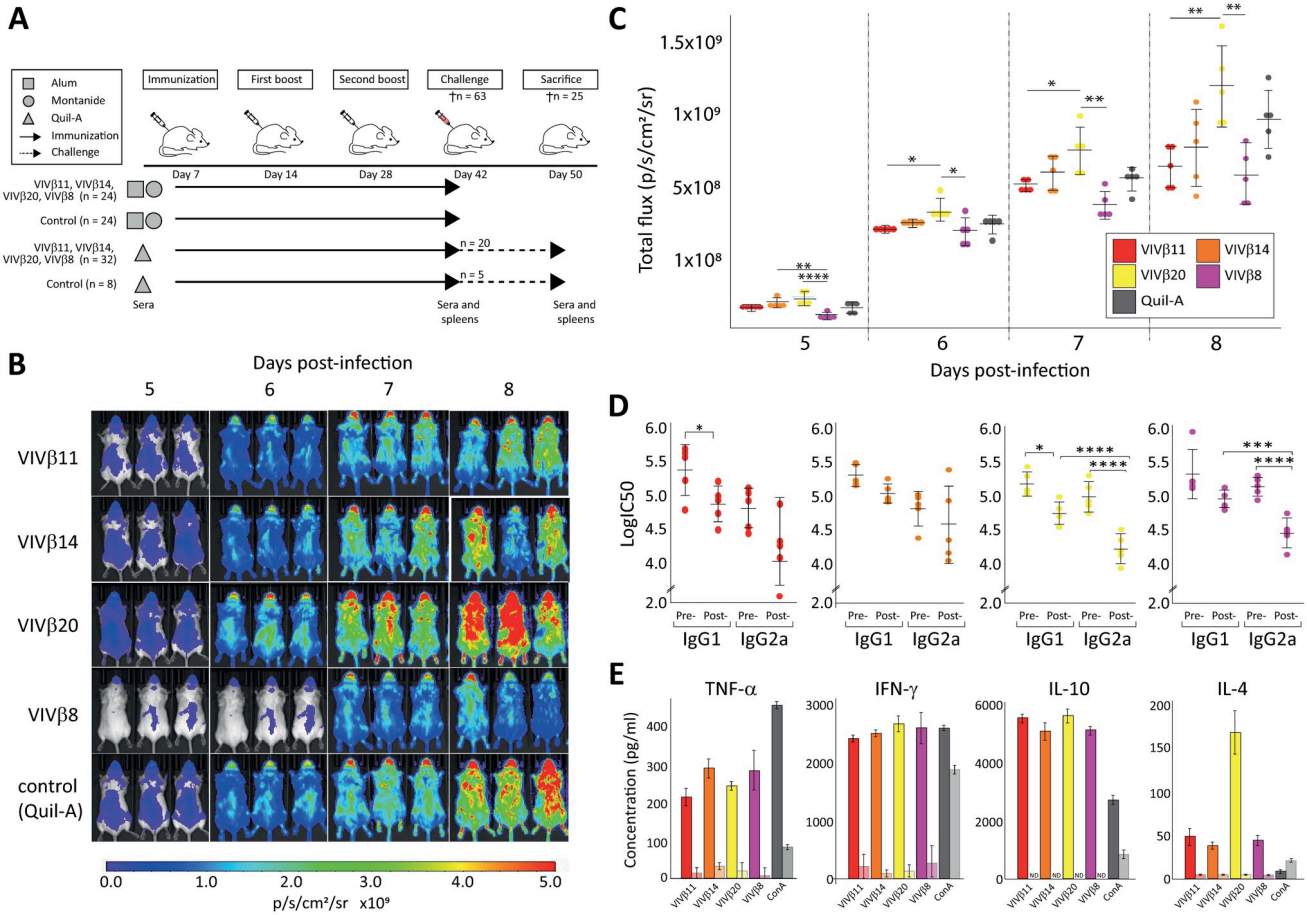
Vaccination and *T*. *vivax* challenge in a murine model. **(A)** Schedule of vaccine immunization and challenge. BALB/c mice received prime immunization followed by two boosts of a protein-in-adjuvant formulation. Each recombinant protein was combined with one of Alum, Montanide ISA 201 VG or Quil-A adjuvants, while the control groups received adjuvants only. Animals were euthanized at day 42 (n = 63) to assess the response to immunization, except for five mice from each vaccinated and control Quil-A-based groups (n = 25) which were challenged with bloodstream-form *T*. *vivax* for a further eight days. **(B)** Vaccine protection against challenge with bioluminescent *T*. *vivax* in BALB/c mice. In vivo imaging of immunized mice with each of four vivaxin antigens co-administrated with Quil-A (n = 5/group). Daily bioluminescent images were collected from 5-8dpi. **(C)** Parasite burden, measured as luminescent values (total flux in photons per second) of luciferase-expressing *T*. *vivax* in challenged mice at 8 dpi. **(D)** Humoral response before and after challenge with *T*. *vivax* in mice vaccinated with four antigens co-administered with Quil-A (n = 8). Comparison of isotype IgG in fully immunized mice at day 42 with challenged mice at 8 dpi. Serum concentration determined by ELISA. **(E)** Cytokine production by splenocytes stimulated in vitro after removal from fully immunized mice at day 42 (left-hand bar, full colour), compared with challenged mice at 8 dpi (right-hand bar, faded). Note that reductions in cytokine concentration post-immunization and post-challenge were significant (P < 0.001) for all antigens but labels are omitted for clarity. Data normality was confirmed with a Shapiro-Wilk test and statistical significance was assessed using a two-tailed ANOVA in R studio. Significance is indicated by asterisks: * (P < 0.05), ** (P < 0.01), *** (P < 0.001), **** (P < 0.0001).

To evaluate the efficacy of vaccination, mouse cohorts were vaccinated with a single vivaxin antigen each co-administered with Quil-A (chosen for its ability to stimulate a protective Th1 response), and were challenged with *T*. *vivax* bloodstream-forms (Fig 3A); parasitaemia was monitored by bioluminescent assay. In all cases, bioluminescence increased over the course of infection (Fig 3B). Before 5dpi, all vaccinated mice showed low parasitaemia levels similar to control groups, and showed no adverse effects of infection. At 6dpi, the VIVβ8 cohort had the lowest parasitaemia with a mean of 2.45x10^8^ p/s, while the other cohorts showed an average luminescence of 2.8x10^8^ p/s. At 8dpi, when luminescence was greatest, the VIVβ20 cohort showed the highest parasitaemia of all groups, significantly greater than VIVβ11 (p = 0.008) and VIVβ8 cohorts (p = 0.002). By 9 dpi, however, all animals were sacrificed as they approached the acceptable limits of adverse welfare affects.

At the end of the experiment, parasite luminescence in control and vaccinated animals was not statistically different (p > 0.05). However, this observation belies notable variation within the VIVβ8 cohort. Three of five VIVβ8 -vaccinated mice showed a delayed onset of parasite proliferation (Fig 3C), and a significant reduction in parasitaemia at 8dpi (p = 0.045). Mean bioluminescence at 8dpi in the partially protected mice was 3.38x10^8^ p/s compared to 7.17x10^8^ p/s in the two unprotected VIVβ8 mice (and 9.8x10^9^ p/s in control mice). Antibody titres correlated positively with this partial protection. This indicates that VIVβ8 co-administered with Quil-A inhibited parasite proliferation in some cases, although without ultimately preventing infection. We repeated the VIVβ8+Quil-A challenge using a larger cohort (n = 15) and an increased dose of antigen (50μg) for vaccination (S7 Fig); this produced a similar but not improved effect. Bioluminescence from vaccinated animals was significantly lower than the control group at 6dpi (p = 0.016). However, there was no beneficial effect by 9dpi, with mice from vaccinated and control groups displaying a mean of 1.45x10^9^ and 1.60x10^9^ p/s, respectively.

After challenge, animals vaccinated with VIVβ14 showed a non-significant reduction in both IgG isotypes, while there was a significant reduction in the IgG1 titration in VIVβ11 and VIVβ20 vaccinated mice (p < 0.01). IgG2a antigen-specific antibody levels also decreased significantly after challenge against VIVβ20 (p < 0.0001) and VIVβ8 (p = 0.0004; Fig 3D). Cytokine levels also displayed pronounced changes after challenge (Fig 3E), irrespective of the antigen involved. IL-10 expression became non-detectable after 8 dpi when compared with pre-vaccination levels (p < 0.001). IL-4 concentrations also decreased significantly after challenge (p < 0.0001). TNF-α and IFN-γ average concentrations against each antigen were reduced significantly (p < 0.0001), representing a reduction of 95% and 92.8% respectively. Unstimulated cells from the adjuvant-only control group showed high cytokine levels indicating that Quil-A alone is able to stimulate their production.

Overall, all four vivaxin antigens were immunogenic, although they differed in the precise balance of immune response elicited, but none was able to protect against acute *T*. *vivax* infection in mouse. Only antigen-4, encoded by *viv-β8*, produced a balanced Th1-Th2 immune response after immunization with Quil-A and went on to inhibit parasite proliferation in some cases. While encouraging, this effect was not observed in all animals, and the balanced immune response was diminished after challenge with the decline of IgG2 titres relative to IgG1.

### Immunofluorescent and electron microscopy localizes VIVβ8 to the whole-cell surface but suggests that it is inaccessible to antibodies

**Fig 4:**
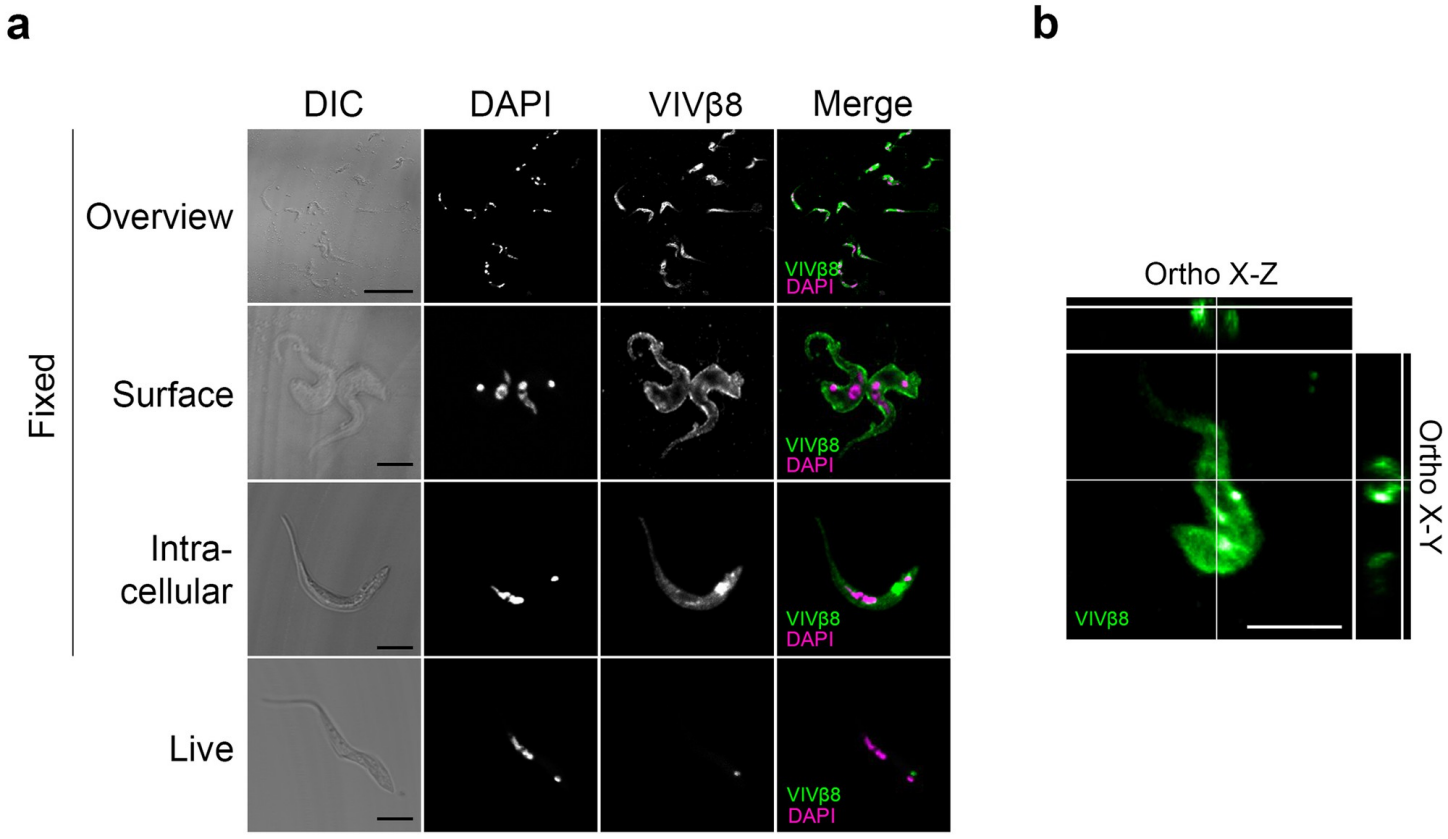
Cellular localization of VIVβ8 protein. **(A)** Representative images of immunofluorescence assays on *T*. *vivax* bloodstream forms fixed in 4% formaldehyde or on live cells. Differential increased contrast (DIC); DAPI DNA counterstain; VIVβ8 (secondary antibody AF555-conjugated) and merged channels. Formaldehyde-fixed cells exhibit three main staining patterns; cell surface (Surface), increasing gradient from anterior to posterior end (Gradient), and intracellular mainly (Intracellular). Scale bars; 5 μm. **(B)** 3D z-stack reconstructions of *T*. *vivax* cells and corresponding orthogonal (X-Z and X-Y) views from the stacks. Orthogonal views note surface localization (circular edges) of VIVβ8 in *T*. *vivax*.

As yet, the cell-surface position of vivaxin is predicted based on amino acid sequence but not proven. If vaccination does not provide protective immunity perhaps this is because VIVβ8 is not surface expressed after all, or not accessible to antibodies. To explore this, we localized VIVβ8 by immunostaining *T*. *vivax* bloodstream forms with anti-VIVβ8 polyclonal antibodies (Fig 4A). When bloodstream form cells were isolated from infected mouse blood; positive stain was associated with the margins of the cell body and flagellum, indicating a specific association with the whole cell surface (Fig 4A, second row). Some cells also showed evidence for intracellular staining, with a noticeable posterior-to-anterior gradient in signal, and a concentrated intensity between the nucleus and kinetoplast (Fig 4A, third row). These observations are not contradictory; endosomes servicing the secretory pathway are known to accumulate at the posterior end of the cell [54], making the intracellular localisation of VIVβ8 consistent with it being trafficked to the cell surface. In fact, surface localisation was confirmed by confocal 3D reconstructions of bloodstream-form cells stained with anti-VIVβ8 antibodies, in where orthogonal views show ring-shaped signal representing the cellular periphery (Fig 4B).

**Fig 5:**
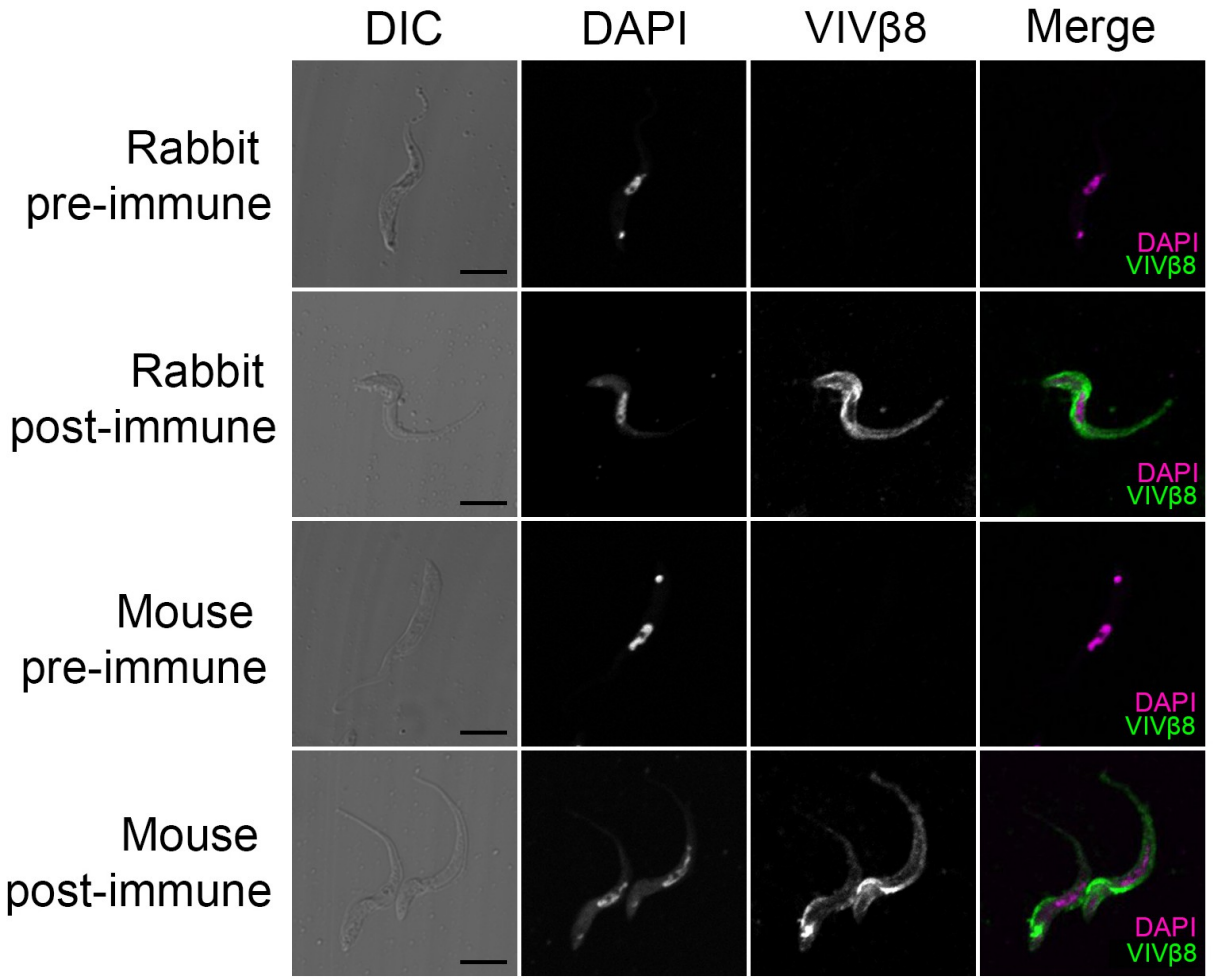
VIVβ8 immunostaining controls. Representative images of *T*. *vivax* bloodstream-form, formaldehyde-fixed cells either probed with pre-immune (pre) or post-immunization (post) antisera from either rabbit or mouse hosts vaccinated with VIVβ8. Only post-immunisation antisera display antibody binding. Scale bars; 5 μm.

To corroborate this result, the post-immune sera of mice and rabbits immunised with recombinant VIVβ8 (residue-residue) was used to stain formaldehyde -fixed bloodstream-stage cells (Fig 5). In both cases, post-immune serum reacted strongly with the entire cell surface and flagellum, resembling the localization found using the polyclonal antibody.

**Fig 6:**
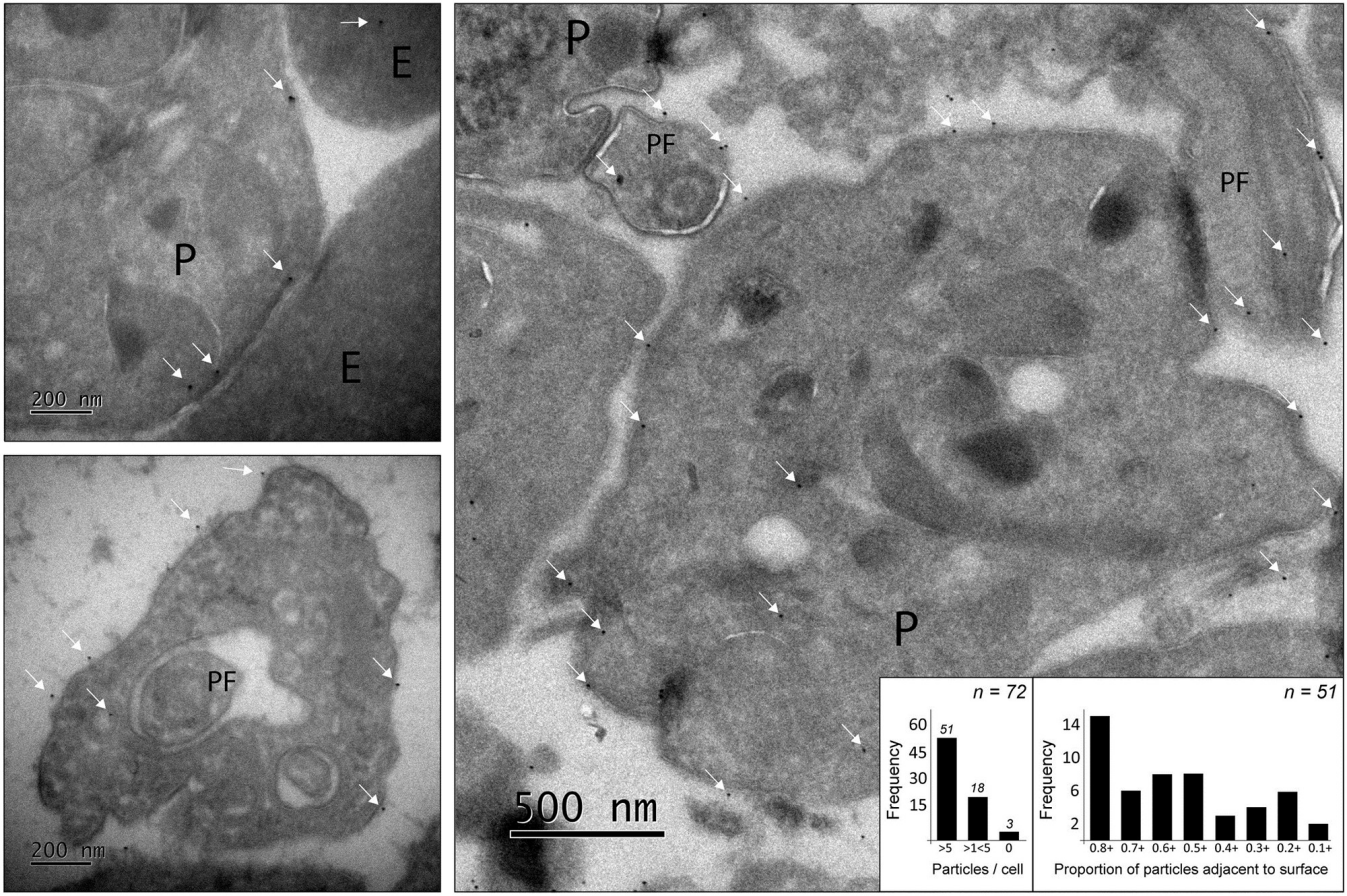
Localisation of VIVβ8 by immunolabelled transmission electron microscopy. Parasites (P) and erythrocytes (E) derived from murine infections were labelled with anti-VIVβ8 polyclonal antibodies. VIVβ8 was localised to the whole parasite cell surface (top and bottom left), and to the parasite flagellum (PF). Anti-VIVβ8-coated gold particles are indicated by white arrows. The right-hand image shows the predominant localisation of VIVβ8 to the surface, although intracellular positions are observed. The graph (inset) shows the proportion of labelled cells and a frequency distribution of the proportion of anti-VIVβ8-coated gold particles found adjacent to the parasite cell membrane of labelled cells.

Greater resolution on the cell surface position of VIVβ8 was achieved with transmission electron microscopy of immunolabelled bloodstream-form cells. Anti-VIVβ8 binding was observed within the cytoplasm but also around the entire cellular periphery (Fig 6), including the flagellar membrane (Fig 6, right). Almost all cells (69/72) were immunolabelled, indicating that VIVβ8 (or, potentially, a closely related protein) was expressed constitutively. For most cells (37/51) that displayed >5 anti-VIVβ8 gold particles, the majority of particles were found adjacent to the cell surface (Fig 6, inset), consistent with the final location of VIVβ8 being on or beyond the plasma membrane.

**Fig 7:**
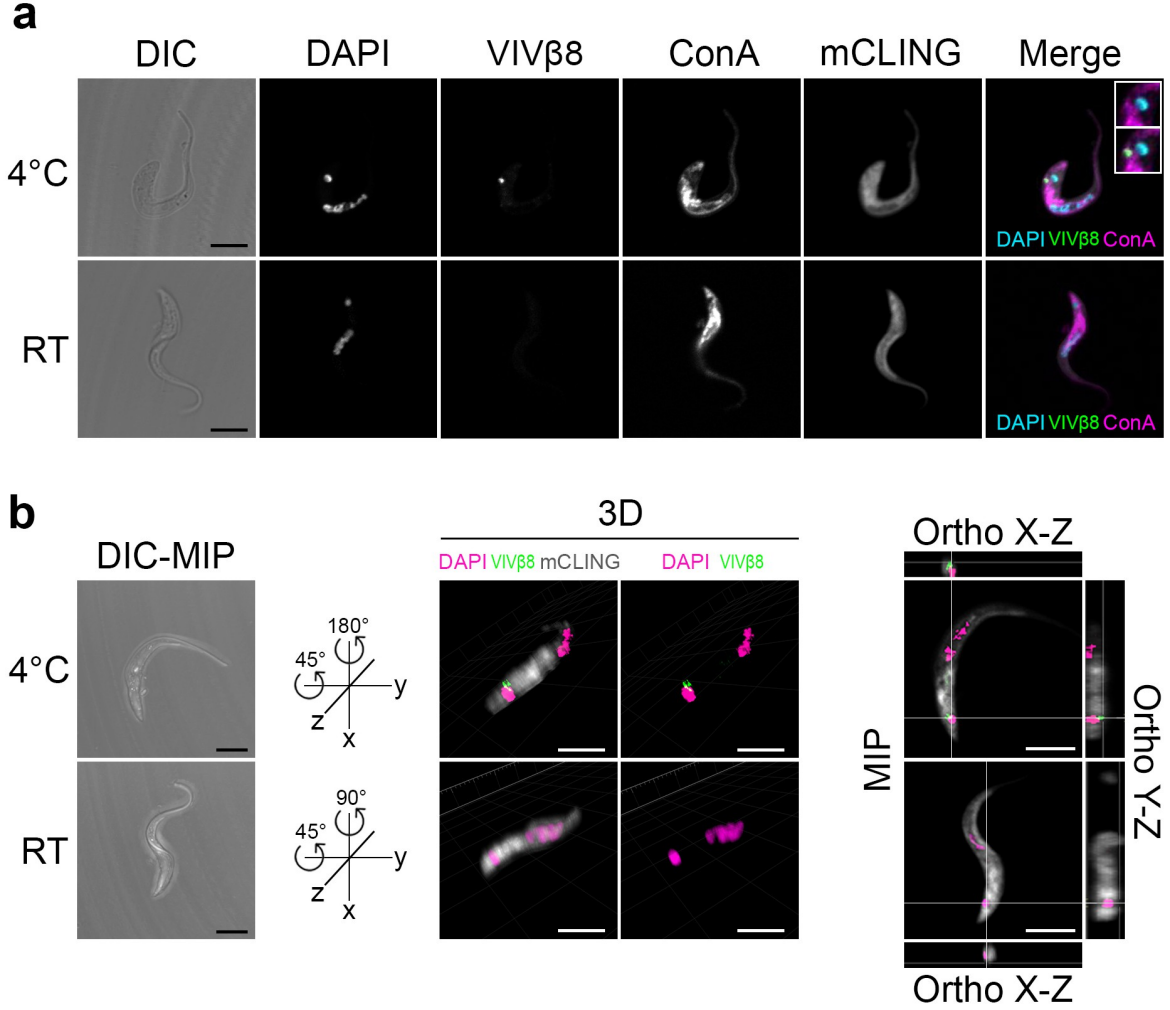
Live immunostaining of VIVβ8 antigen in native cells. **(A)** Live *T*. *vivax* bloodstream forms were immunostained either at 4°C to halt the secretory pathway or at room temperature (RT) to preserve it active. DIC; DAPI; VIVβ8 (secondary AF555-conjugated antibody); concanavalin A (ConA) FITC-conjugated lectin ER counterstain; mCLING unspecific staining and merged channels. Close-ups in merge channel show the flagellar pocket (end of ER, next to kDNA) without (top) or with (bottom) VIVβ8 green signal. Scale bars; 5 μm. **(B)** 3D localization of VIVβ8 in live cells. Representative cells immunostained at 4°C or RT were 3D reconstructed in DIC-maximum intensity projection (DIC-MIP, left) and rotated to equivalent positions in the space to display VIVβ8 next to the kDNA only in 4°C cells (middle). MIP fluorescence projections (right) and orthogonal views. Scale bars; 5 μm.

Thus, the lack of protection afforded by VIVβ8 vaccination is not due to an intracellular position. Yet, it is possible that fixation of cells affects the disposition of vivaxin on the cell surface. To assess the accessibility of vivaxin epitopes in a native setting, we performed immunostaining on live cells at room temperature (RT) and at 4°C to arrest cell endocytosis (Fig 7A). Unlike in fixed cells, those probed at RT were not stained. However, 4°C-incubated cells localised anti-VIVβ8 exclusively to the flagellar pocket, confirmed by its position next to the kDNA in 3D reconstructions (Fig 7B). As in a previous study [55], we interpret this as evidence for endocytosis. At RT, antibody-bound VIVβ8 is rapidly cleared, but at 4°C, the antibody is not removed and accumulates where VIVβ8 epitopes are exposed.

Thus, VIVβ8 is likely expressed on the plasma membrane, but there is a disparity between immunostaining of formaldehyde-fixed cells, which establishes staining of the whole cell surface, and of live cells, which indicates that VIVβ8 localises to the flagellar pocket only. This can be explained by epitope availability, if VIVβ8 is distributed across the whole cell membrane but obscured from antibody binding in its native state, perhaps by other proteins in the surface glycocalyx, and only revealed in its full distribution after epitopes are exposed by formaldehyde fixation.

Finally, it is worth noting that we also observed anti-VIVβ8 staining on red blood cells after formaldehyde fixation of *T*. *vivax*-infected mouse blood (S8a Fig). Although the orthogonal view (S8b Fig) might suggest that the stain is intracellular, but since mature red blood cells are not thought to endocytose, we suggest that the observed pattern reflects the concave cell, and that the stain localises in the centre of the bi-concave cell area. This pattern affected all red cells we examined. It is unclear whether VIVβ8 is secreted actively by *T*. *vivax* to target the host erythrocytes, or transferred accidentally after parasite cell lysis.

## Discussion

We examined the antibody responses of naturally and experimentally infected hosts to diverse TvCSP to identify immunogens that could become the basis for a *T*. *vivax* vaccine. Immunoprofiling of host serum showed that one particular protein family (Fam34), now known as vivaxin, includes consistently the most immunogenic proteins among the set we tested. In fact, the prominence of vivaxin was implied in a previous study by Fleming et al. [56]; they identified two related proteins (TvY486_0019690/0045500) of particular immunogenicity in *T*. *vivax* infections and demonstrated their diagnostic potential. These two proteins can now be identified as vivaxin members (VIVβ26 and VIVβ27), part of a large gene family encoding transmembrane glycoproteins with a conserved primary structure but diverse expression profiles and population genetic dynamics. Thus, not all vivaxin genes are equally good candidate antigens; we focused on one gene with minimal polymorphism (*viv-β8*) that was among the most immunogenic and highly expressed, and confirmed its expression across the cell body and flagellar membranes.

While VIVβ8 elicits a strong, balanced humoral and cellular immune response with Quil-A and significantly reduces parasite burden in some mice by delaying peak parasitaemia, animals were not protected from acute fatal disease. In fact, the experiment followed a familiar pattern, with reduced antibody titres and pro-inflammatory cytokine concentrations after challenge underscoring the impact of infection induced immunomodulation. Reduction of IgG1 and IgG2a antibody titres before and after challenge was observed previously in vaccinated cattle challenged with both *T*. *vivax* [57] and *T*. *congolense* [58], as well as vaccinated mice challenged with *T*. *brucei* [21]. These observations could be explained by a decline in total circulating WBCs, which is mirrored in the spleen where normal tissue architecture is lost and parasite-driven necrosis becomes prominent [59]. This loss of white pulp organization occurs in mice where homeostasis of both bone marrow and splenic B-cells is perturbed [60]. Another possible explanation could be the formation of immune complex of specific antibodies with the parasite antigens making it difficult to measure IgGs solely in circulation [61–62]. Reduction of antigen-stimulated cytokine levels observed after 8 dpi is not conducive to parasite control, since IFN-γ is associated with resistance to African trypanosomes [51–53] and TNF-α was shown to be essential to controlling *T*. *vivax* infection in mice [63]. Importantly, parasite-driven necrosis destroying host B-cells and leading to mortality, as above, occurs independent of TNF-α [64], indicating that mortality arises from parasite pathology rather than immunopathology, and suggesting that the protective effects of vaccine driven parasite-specific cytokines are more protective than harmful. These changes in both the IgG1/IgG2a ratio and the cytokine profiles after challenge indicate a transition from a Th1-type to Th2-type response, which is a typical feature of uncontrolled infections in trypanosomatids [65–69] and is observed in chronically naturally-infected cattle [70]. Ultimately, vaccine driven responses may require biasing towards the Th1 spectrum.

Thus, after vaccination with VIVβ8 infection took its normal course. We should recognize that the lack of protection could be due to limitations in our approach, such as the use of a murine model in which parasite virulence is atypically high, or the use of expression of recombinant proteins in a mammalian cell line, although a positive result has been obtained for another *T*. *vivax* antigen using the same experimental model [23]. Assuming that the lack of protection is not an artifact, it could be that vivaxin epitopes are hidden in situ or that bound vivaxin is removed via endocytosis under physiological conditions. Immunofluorescent microscopy of fixed trypomastigotes using both purified recombinant antibodies (Fig 4) and post-immune serum (Fig 5), as well as electron microscopy (Fig 6), indicate that VIVβ8 is located across the entire cell surface, and so, a uniform component of the glycocalyx alongside the VSG. However, when antibodies were applied to live parasites they fail to bind, except when the cells are cooled, and then only to the flagellar pocket (Fig 7), indicating that these vivaxin proteins are rapidly removed from the surface by endocytosis or largely concealed in some way, though not by a VSG monolayer since structural characterization of surface receptors in *T*. *brucei* has indicated that the VSG layer does not physically conceal other surface proteins [71–72] and, in any case, vivaxin predicted proteins are on average at least equally large as a typical *T*. *vivax* VSG (~450 amino acids).

If vivaxin epitopes are inaccessible in vivo, we must explain the strong and consistent serological response of both naturally and experimentally-infected animals to multiple vivaxin proteins. The strength of the serological response perhaps reflects the abundance and conservation of vivaxin, since we linked the most abundant and least polymorphic transcripts to the strongest immunogens. However, vivaxin may be secreted and eliciting antibodies after cleavage of the extracellular domain from the cell surface, or else, the antibody response may be directed primarily at dead and lysed parasites. Such responses would not affect live, circulating parasites if the protein remained concealed on their surface.

Although VIVβ8 may not elicit protective immunity, our phylogenetic analysis reveals that two antigens that were effective in a previous study, IFX and V31 [23], belong to the vivaxin gene family. We now realize the IFX (VIVβ1) is also among the most strongly expressed and structurally conserved vivaxin proteins (although not as much as VIVβ8). V31 (VIVα18) had a partially protective effect in mice, but is much more polymorphic [23]. Curiously, *viv-β1* (encoding IFX) adopts a unique position within the phylogeny, as the sister lineage to all other *β*-vivaxin. This topology is highly robust but nonetheless odd, because *viv-β1* is much longer than other *β*-vivaxin and noticeably divergent (note the length of the *viv-β1* branch). It is tempting to speculate from the strong conservation and divergent structure of VIVβ1 that this protein performs a distinct, non-redundant function among vivaxin proteins, a function that evidently exposes it to antibodies unlike many of its paralogs. Note that while VIVβ8 localizes across the cell body and flagellum, VIVβ1 was restricted to regions of the flagellar membrane [23]. Thus, among the 124 (and likely more) vivaxin paralogs there is great potential for reliably immunogenic and protective antigens; yet this study reveals substantial variability in structure and antigenic properties, even among closely related gene copies, such that not all vivaxin proteins will make good antigens.

Besides its potential for subunit vaccines, the discovery of vivaxin has implications for host-parasite interactions. The protein architecture of the *T*. *vivax* cell surface is not well characterised, partly because attention is more typically focused on the human pathogen *T*. *brucei*, but also because there are few research tools (e.g., in vitro cell culture, reverse genetics, mouse infection model) developed for *T*. *vivax* [73]. Yet, recent results and historical anecdote suggest that the *T*. *vivax* cell surface is quite different to the uniform and pervasive VSG monolayer of *T*. *brucei*. Vickerman considered the *T*. *vivax* surface coat to be less dense than other species [74–75]. The *T*. *vivax* genome contains hundreds of species-specific and non-VSG genes [24–25,45]. Greif et al. (2013) showed that only 57% of surface-protein encoding transcripts during *T*. *vivax* mouse infections encoded VSG (compared to 98% of *T*. *brucei* bloodstream-stage surface-protein encoding transcripts) and that the remainder belonged largely to *T*. *vivax*-specific genes [49]. This study shows that vivaxin must be a major contributor to this difference between *T*. *vivax* and *T*. *brucei* surfaces.

It follows that, with a different cell surface architecture, *T*. *vivax* may interact with hosts in a different way, dependent on what the function(s) of vivaxin might be. Other trypanosome surface proteins are variant antigens (e.g. VSG [76]), immunomodulators in other ways (e.g. trans-sialidases [77]), scavenge nutrients (e.g. transferrin and HpHb receptors [78–79]) or sense the host environment (e.g. adenylate cyclases [80–81]). Various molecular evolutionary aspects, (i.e. strong purifying selection, low polymorphism, maintenance of gene orthology), as well as the absence of monoallelic expression, indicate that vivaxin are not variant antigens. However, other functions in immunomodulation or pathogenesis are plausible. Attachment between erythrocytes and the *T*. *vivax* cell surface has been observed in sheep and is associated with mechanical and biochemical damage to red blood cells that contributes to pathology [82]. The secretion (perhaps passively through cell lysis, or actively via exosomes) of VIVβ8 and its adhesion to erythrocytes (S8 Fig) could suggest that vivaxin contributes to cytoadhesion, possibly leading to parasite sequestration in tissue capillaries as an immune evasion strategy. During *T*. *brucei* infections, VSG and other trypanosome surface proteins are deposited on the surface of murine erythrocytes [83]; in this case, secretion is mediated by parasite exosomes, fusion of which alters the erythrocyte cell membrane, leading to erythrophagocytosis and likely contributing to anaemia [83]. Future studies should consider whether vivaxin is actively secreted into the bloodstream in a similar way.

Perhaps the only aspect of vivaxin function we can predict presently is that it will be multifarious. Differences in length among subfamilies will translate into distinct tertiary protein structures, while consistent differences in expression profile suggest that some vivaxin genes are ‘major forms’, while other paralogs appear to be non-functional, and a few may be expressed beyond the bloodstream-stage. Population genetics show that vivaxin genes evolve under a range of selective conditions, from strongly negative (i.e. functionally essential and non-redundant), to neutral (i.e. redundant), and positive (engaged in antagonistic host interactions?). Coupled with the evolutionary stability of these features, (that is, individual vivaxin genes are found in orthology across *T*. *vivax* strains rather than recombining or being gained and lost frequently), this is evidence for functional differentiation and non-redundancy within the gene family.

Vivaxin represents a major component of the *T*. *vivax* surface coat, quite distinct from VSG, and includes proven vaccine targets, and many more potential targets. The molecular evolution of vivaxin implies that the paralogous gene copies lack the dynamic variability and redundancy of variant antigens, but instead perform multiple functions, and at least some genes may be essential. The discovery of this highly immunogenic and abundant protein family has important implications for how we approach AAT caused by *T*. *vivax* because, although it may yet be found in other Salivarian trypanosomes, it is certainly not found in *T*. *brucei*. Thus, it challenges the adequacy of *T*. *brucei* as a model for AAT, given the different qualities of their surface architectures, while posing new therapeutic opportunities and new questions about the roles vivaxin has in host interaction, immune modulation and disease.

## Supporting information

S1 Fig Peptide microarray slide design. The diagram shows the 600 spots of the microarray (scale at edge), with each cell corresponding to a 15-mer peptide, printed in duplicate, belonging to one of 63 *Trypanosoma vivax* proteins, or a control peptide. The cells are shaded to identify the *T*. *vivax* cell surface phylome (TvCSP) to which each non-control peptide belongs [24]. Twenty-one proteins do not belong to multi-copy families (‘Single-copy’), but are still predicted to have cell surface expression.
(DOCX)

S2 Fig Predicted secondary protein structures for six vivaxin genes. The six genes include those four encoding antigens 1–4 identified in this study and expressed in recombinant form (*viv-β11*, *viv-β14*, *viv-β20* and *viv-β8*), as well as two others encoding candidate antigens from another study (*viv-β1* and *viv-α18*; [23]) for comparison. Protein secondary structures were inferred from amino acid sequences using PredictProtein [41]: alpha helices (red), transmembrane helix (purple), disordered region (green). The solvent accessibility of each position is also indicated: accessible (blue) and buried (yellow). N- and O-linked glycosylation sites were predicted using ModPred [42] and are indicated by red and orange arrows respectively. The position of linear b-cell epitopes inferred from the TvCSP peptide microarray are indicated by grey bars at the bottom of each diagram (the range of positions in the amino acid sequence is given).
(DOCX)

S3 Fig Recombinant expression of four vivaxin proteins using a mammalian expression system. A) Normalization of antigen 1 (VIVβ11) protein using two-fold serial dilutions. B) Normalization of antigens 2–4 (VIVβ14, VIVβ20 and VIVβ8). The concentration of biotinylated proteins was determined by ELISA. C) Purified vivaxin proteins were resolved by SDS-PAGE on a 12% NUPAGE SDS/polyacrylamide gel (under reducing conditions) and stained with Sypro orange. M: molecular mass marker. The gel showed a prominent band with apparent molecular mass of 50kDa for each recombinant protein. The antigens have a predicted molecular mass of 34-39kDa based on amino acid sequence alone, i.e. before glycosylation. Based on the extinction coefficient calculation, the purified proteins had a concentration of 4.3mg/mL (antigen 1; VIVβ11), 5.1mg/mL (antigen 2; VIVβ14), 9.8mg/mL (antigen 3; VIVβ20) and 2.5 mg/mL (antigen 4; VIVβ8). Note that the weaker, higher molecular mass bands that were also observed for all antigens are likely due to co-purifying proteins from the tissue culture supernatant. Smearing in the bands is probably due to variation in glycosylation. Almost all glycoprotein preps are a complex mixture of different glycoforms, which vary in the precise occupation of N-linked glycosylation sites as well as the actual glycan attached at each site.
(DOCX)

S4 Fig Antibody titres after immunization. Both IgG1 and IgG2a-specific antibody titres in mice immunized with four different antigens are compared with two negative controls (pre-immune sera and adjuvant-only mice). There is a consistent response for all antigens regardless of the adjuvant used. However, adjuvant choice has a significant effect on antibody titres. Montanide produced significantly higher IgG1 levels than either Alum or Quil-A when applied with VIVβ11, VIVβ14 and VIVβ20, but, there was no difference in IgG1 titre between adjuvants when VIVβ8 was used. In contrast, Quil-A produced significantly higher IgG2a titres than either Montanide or Alum when applied with all antigens. Data normality was confirmed with a Shapiro-Wilk test and statistical significance was assessed using a one-tailed ANOVA in R studio. Significance is indicated by asterisks: * (P < 0.05), *** (P < 0.001), **** (P < 0.0001).
(DOCX)

S5 Fig Titres of IgG1 and IgG2a isotypes in infected cattle against four antigens, measured by indirect ELISA. IgG1 and IgG2a specific antibody titres were measured using two-fold serial dilutions in naturally infected (Cameroon and Kenya) and experimentally infected cattle (Brazil). Antibody levels were also measured in a group of UK cattle, which served as negative controls. IgG1 showed higher levels when compared to IgG2a for both natural and experimental infections with *T*. *vivax*. Each graph shows the antibody levels of individual serum, the geometric mean of each group, and the 95% confidence interval. Data normality was confirmed with a Shapiro-Wilk test and statistical significance was assessed using a one-tailed ANOVA in R studio. Significance is indicated by asterisks: **** (P < 0.0001).
(DOCX)

S6 Fig Cytokine expression after immunization compared for different adjuvants. Concentrations of four cytokines (TNF-α, IFN-γ, IL-10 and IL-4) were measured in ex vivo mouse splenocyte cultures after stimulation with each vivaxin antigen, co-administrated with one of three adjuvants. Concanavalin A was applied as a positive control. Stimulation with adjuvant only was applied as a negative control. A cross (+) denotes that a value could not be determined. Data normality was confirmed with a Shapiro-Wilk test and statistical significance was assessed using a one-tailed ANOVA in R studio. Significance is indicated by asterisks: ** (P < 0.01), *** (P < 0.001), **** (P < 0.0001).
(DOCX)

S7 Fig VIVβ8 vaccination and challenge experiment in BALB/c mice repeated with a larger cohort (n = 15/group) and antigen dose (50μg); protocol as described in methods. **A.** Luciferase intensity from VIVβ8+Quil-A-vaccinated animals was significantly lower than the adjuvant-only control group at 6 dpi (p = 0.016), with means of 1.32x108 and 1.71x108 p/s respectively. On subsequent days, there were no significant differences between luminescence values of vaccinated and control groups. **B.** Kaplan-Meir survival curve (%) of both groups during the course of infection. **C.** Bioluminescence values from VIVβ8-vaccinated and control animals compared. **D.** Isotype IgG profiling in challenged animals culled at 8 and 9 dpi. **E.** Cytokine levels in challenged animals culled at 8 and 9 dpi. There were no significant changes in TNF-α, IFN-γ and IL-10 concentrations between 8 dpi and 9 dpi. There was a significant rise in IL-4 concentration, with undetectable values at 8 dpi and an average concentration of 7.83pg/ml at 9 dpi (p = 0.028). The comparison between 8 dpi and 9 dpi also showed pronounced changes in IL-10 and IL-4 levels in the control group stimulated with ConA (p = 3.40E-04 and p = 1.78E-04 for IL-10 and IL-4, respectively). In all cases, cytokine concentration from splenocytes stimulated with VIVβ8 was lower than the control group stimulated with ConA, except for IL-4 levels at 9 dpi. Data normality was confirmed with a Shapiro-Wilk test and statistical significance was assessed using a one-tailed ANOVA (panel D) or paired t-test (panel E) in R studio. Significance is indicated by asterisks: * (P < 0.05), ** (P < 0.01), *** (P < 0.001), **** (P < 0.0001).
(DOCX)

S8 Fig Cellular localization of VIVβ8 antigen on the surface of murine erythrocytes. **(A)** Localization of VIVβ8 and the unspecific surface counterstain mCLING in red blood cells (RBC) from *T*. *vivax*-infected mice. Representative images of RBC stained with either pre-immune or post-immune rabbit polyclonal antisera. Middle row shows the major localization pattern of VIVβ8 in RBC; protein accumulates in the central concave surface. Bottom row shows an example of leaking RBC. Differential increased contrast (DIC); DAPI DNA counterstain; VIVβ8 (secondary antibody AF555-conjugated) and merged channels. Scale bars; 5 μm. **(B)** 3D z-stack reconstructions of mouse erythrocyte cells and corresponding orthogonal (X-Z and X-Y) views from the stacks. Orthogonal views reflect the accumulation of VIVβ*8* signal at the inner concave cell membrane. Scale bars; 5 μm.
(DOCX)

S1 Table Raw response intensity (RRI) values for peptide array spots when assayed with infected serum and results of limma analysis. The table contains all data obtained from the peptide array assay, showing the peptide sequence and parent gene for each of 600 spots on the array. Where appropriate, the gene family is noted, or the gene is marked as ‘single-copy’ (‘SCG’) otherwise. The mean RRI value when assayed with infected serum (averaged across two duplicate spots) is followed by a statistical comparison (t-test) with the uninfected control (log_2_ fold-change in RRI and adjusted P-value).
(XLSX)
