# Supplementary material for: Vivaxin genes encode highly immunogenic, non-variant antigens on the Trypanosoma vivax cell-surface
Source: PLoS Negl Trop Dis. 2022 Sep 21;16(9):e0010791. doi: 10.1371/journal.pntd.0010791 (PMC9529106; doi:10.1371/journal.pntd.0010791)
Supplement: S1 Fig — The diagram shows the 600 spots of the microarray (scale at edge), with each cell corresponding to a 15-mer peptide, printed in duplicate, belonging to one of 63 Trypanosoma vivax proteins, or a control peptide. The cells are shaded to identify the T. vivax cell surface phylome (TvCSP) to which each non-control peptide belongs [24]. Twenty-one proteins do not belong to multi-copy families (‘Single-copy’), but are still predicted to have cell surface expression. (DOCX) [file pntd.0010791.s001.docx]

**
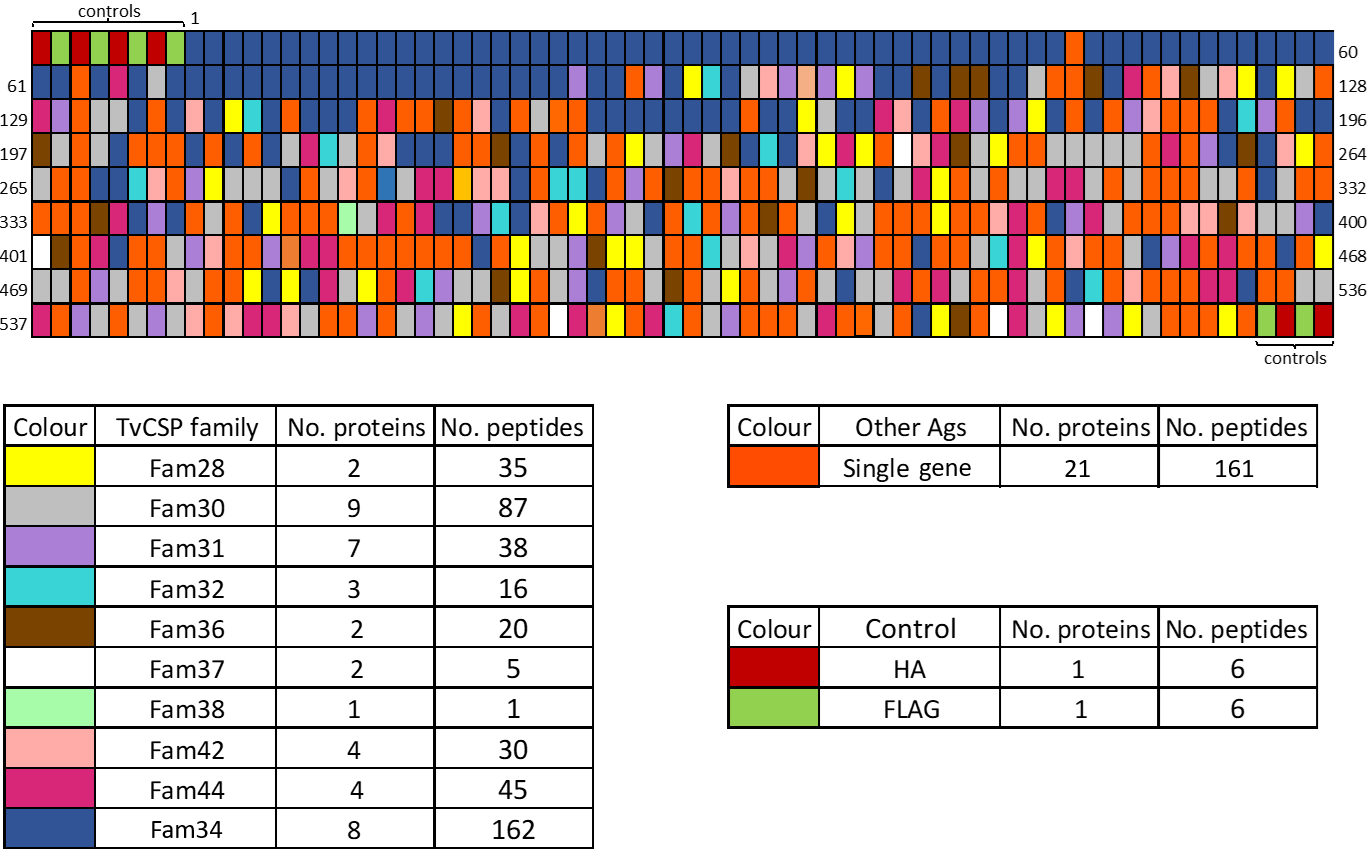
**

**S1 Fig. Peptide microarray slide design.** The diagram shows the 600 spots of the microarray (scale at edge), with each cell corresponding to a 15-mer peptide, printed in duplicate, belonging to one of 63 *Trypanosoma vivax* proteins, or a control peptide. The cells are shaded to identify the *T. vivax* cell surface phylome (TvCSP) to which each non-control peptide belongs [1]. Twenty-one proteins do not belong to multi-copy families (‘Single-copy’), but are still predicted to have cell surface expression.

**Reference:**

1. Jackson AP, Allison HC, Barry JD, Field MC, Hertz-Fowler C, Berriman M. A Cell-surface Phylome for African Trypanosomes. PLoS Negl. Trop. Dis. 2013; 7(3):e2121.
