# Supplementary material for: Vivaxin genes encode highly immunogenic, non-variant antigens on the Trypanosoma vivax cell-surface
Source: PLoS Negl Trop Dis. 2022 Sep 21;16(9):e0010791. doi: 10.1371/journal.pntd.0010791 (PMC9529106; doi:10.1371/journal.pntd.0010791)
Supplement: S2 Fig — The six genes include those four encoding antigens 1–4 identified in this study and expressed in recombinant form (viv-β11, viv-β14, viv-β20 and viv-β8), as well as two others encoding candidate antigens from another study (viv-β1 and viv-α18; [23]) for comparison. Protein secondary structures were inferred from amino acid sequences using PredictProtein [41]: alpha helices (red), transmembrane helix (purple), disordered region (green). The solvent accessibility of each position is also indicated: accessible (blue) and buried (yellow). N- and O-linked glycosylation sites were predicted using ModPred [42] and are indicated by red and orange arrows respectively. The position of linear b-cell epitopes inferred from the TvCSP peptide microarray are indicated by grey bars at the bottom of each diagram (the range of positions in the amino acid sequence is given). (DOCX) [file pntd.0010791.s002.docx]

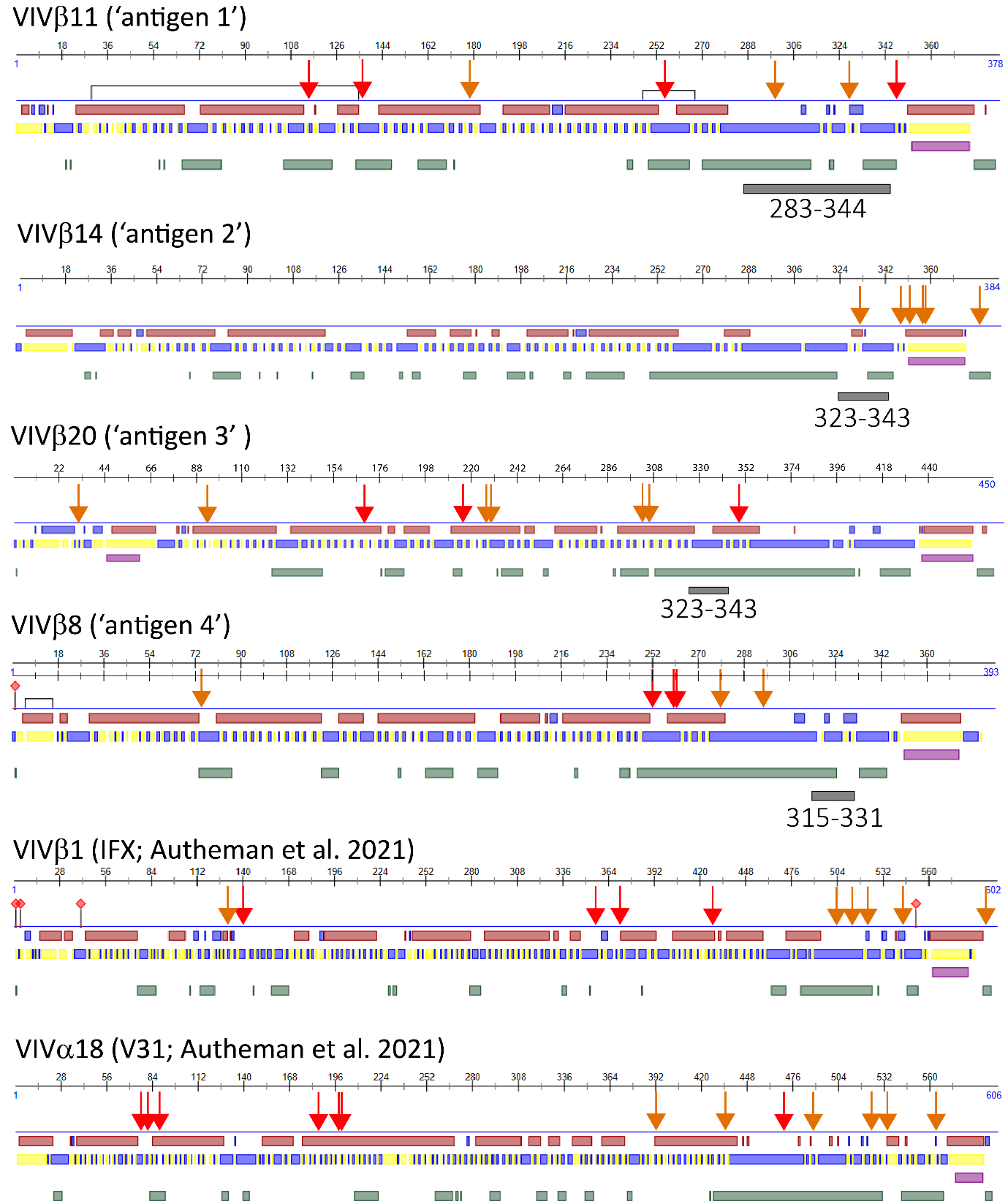


**S2 Fig. Predicted secondary protein structures for six vivaxin genes.** The six genes include those four encoding antigens 1-4 identified in this study and expressed in recombinant form (*viv-β11, viv-β14*, *viv-β20* and *viv-β8*), as well as two others encoding candidate antigens from another study (*viv-β1* and *viv-α18*; [23]) for comparison. Protein secondary structures were inferred from amino acid sequences using PredictProtein [1]: alpha helices (red), transmembrane helix (purple), disordered region (green). The solvent accessibility of each position is also indicated: accessible (blue) and buried (yellow). N- and O-linked glycosylation sites were predicted using ModPred [2] and are indicated by red and orange arrows respectively. The position of linear b-cell epitopes inferred from the TvCSP peptide microarray are indicated by grey bars at the bottom of each diagram (the range of positions in the amino acid sequence is given).

**References:**

1. Yachdav G, Kloppmann E, Kajan L, Hecht M, Goldberg T, Hamp T, et al. PredictProtein–-an open resource for online prediction of protein structural and functional features. Nuc Acid Res. 2014; gku366.
2. Pejaver V, Hsu W-L, Xin F, Dunker AK, Uversky VN, Radivojac P. 2014. The structural and functional signatures of proteins that undergo multiple events of post-translational modification Prot Sci. 2014; 23(8):1077-1093.
