# Supplementary material for: Vivaxin genes encode highly immunogenic, non-variant antigens on the Trypanosoma vivax cell-surface
Source: PLoS Negl Trop Dis. 2022 Sep 21;16(9):e0010791. doi: 10.1371/journal.pntd.0010791 (PMC9529106; doi:10.1371/journal.pntd.0010791)
Supplement: S3 Fig — A) Normalization of antigen 1 (VIVβ11) protein using two-fold serial dilutions. B) Normalization of antigens 2–4 (VIVβ14, VIVβ20 and VIVβ8). The concentration of biotinylated proteins was determined by ELISA. C) Purified vivaxin proteins were resolved by SDS-PAGE on a 12% NUPAGE SDS/polyacrylamide gel (under reducing conditions) and stained with Sypro orange. M: molecular mass marker. The gel showed a prominent band with apparent molecular mass of 50kDa for each recombinant protein. The antigens have a predicted molecular mass of 34-39kDa based on amino acid sequence alone, i.e. before glycosylation. Based on the extinction coefficient calculation, the purified proteins had a concentration of 4.3mg/mL (antigen 1; VIVβ11), 5.1mg/mL (antigen 2; VIVβ14), 9.8mg/mL (antigen 3; VIVβ20) and 2.5 mg/mL (antigen 4; VIVβ8). Note that the weaker, higher molecular mass bands that were also observed for all antigens are likely due to co-purifying proteins from the tissue culture supernatant. Smearing in the bands is probably due to variation in glycosylation. Almost all glycoprotein preps are a complex mixture of different glycoforms, which vary in the precise occupation of N-linked glycosylation sites as well as the actual glycan attached at each site. (DOCX) [file pntd.0010791.s003.docx]

C.

Antigen-2

Antigen-3

Antigen-4

**Antigen:**

**M 1 2 3 4**

Antigen-1


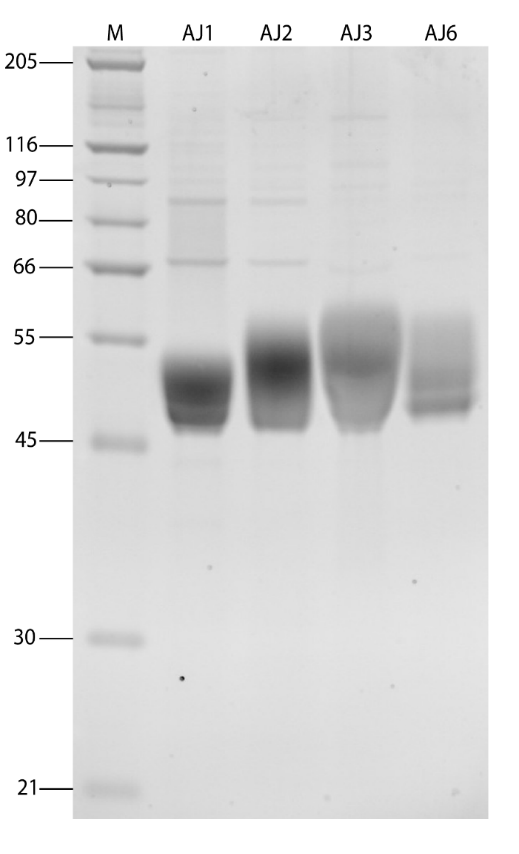

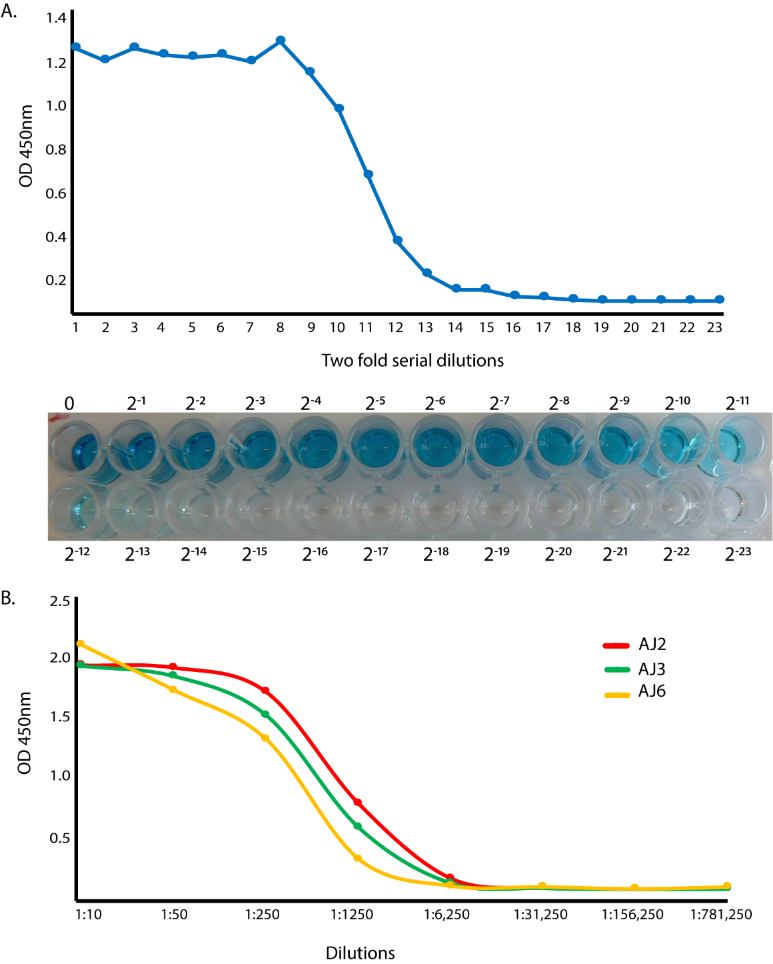

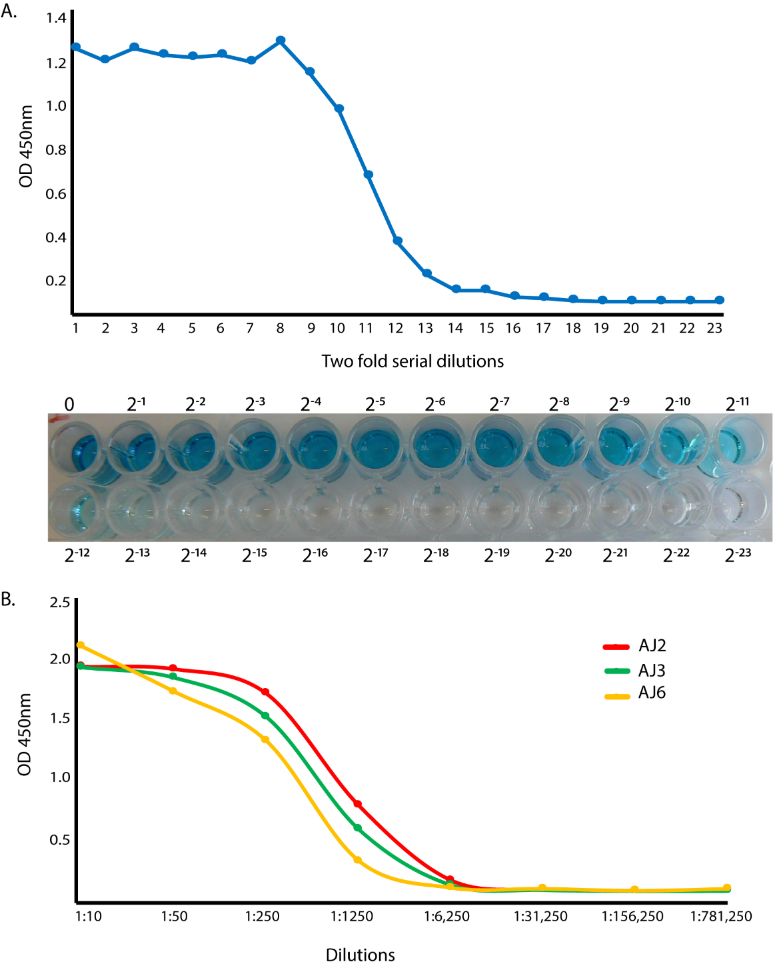


**S3 Fig. Recombinant expression of four vivaxin proteins using a mammalian expression system.** A) Normalization of antigen 1 (VIVβ11) protein using two-fold serial dilutions. B) Normalization of antigens 2-4 (VIVβ14*,* VIVβ20 and VIVβ8). The concentration of biotinylated proteins was determined by ELISA. C) Purified vivaxin proteins were resolved by SDS-PAGE on a 12% NUPAGE SDS/polyacrylamide gel (under reducing conditions) and stained with Sypro orange. M: molecular mass marker. The gel showed a prominent band with apparent molecular mass of 50kDa for each recombinant protein. The antigens have a predicted molecular mass of 34-39kDa based on amino acid sequence alone, i.e. before glycosylation. Based on the extinction coefficient calculation, the purified proteins had a concentration of 4.3mg/mL (antigen 1; VIVβ11), 5.1mg/mL (antigen 2; VIVβ14), 9.8mg/mL (antigen 3; VIVβ20) and 2.5 mg/mL (antigen 4; VIVβ8). Note that the weaker, higher molecular mass bands that were also observed for all antigens are likely due to co-purifying proteins from the tissue culture supernatant. Smearing in the bands is probably due to variation in glycosylation. Almost all glycoprotein preps are a complex mixture of different glycoforms, which vary in the precise occupation of N-linked glycosylation sites as well as the actual glycan attached at each site.
