# Supplementary material for: Vivaxin genes encode highly immunogenic, non-variant antigens on the Trypanosoma vivax cell-surface
Source: PLoS Negl Trop Dis. 2022 Sep 21;16(9):e0010791. doi: 10.1371/journal.pntd.0010791 (PMC9529106; doi:10.1371/journal.pntd.0010791)
Supplement: S4 Fig — Both IgG1 and IgG2a-specific antibody titres in mice immunized with four different antigens are compared with two negative controls (pre-immune sera and adjuvant-only mice). There is a consistent response for all antigens regardless of the adjuvant used. However, adjuvant choice has a significant effect on antibody titres. Montanide produced significantly higher IgG1 levels than either Alum or Quil-A when applied with VIVβ11, VIVβ14 and VIVβ20, but, there was no difference in IgG1 titre between adjuvants when VIVβ8 was used. In contrast, Quil-A produced significantly higher IgG2a titres than either Montanide or Alum when applied with all antigens. Data normality was confirmed with a Shapiro-Wilk test and statistical significance was assessed using a one-tailed ANOVA in R studio. Significance is indicated by asterisks: * (P < 0.05), *** (P < 0.001), **** (P < 0.0001). (DOCX) [file pntd.0010791.s004.docx]

**
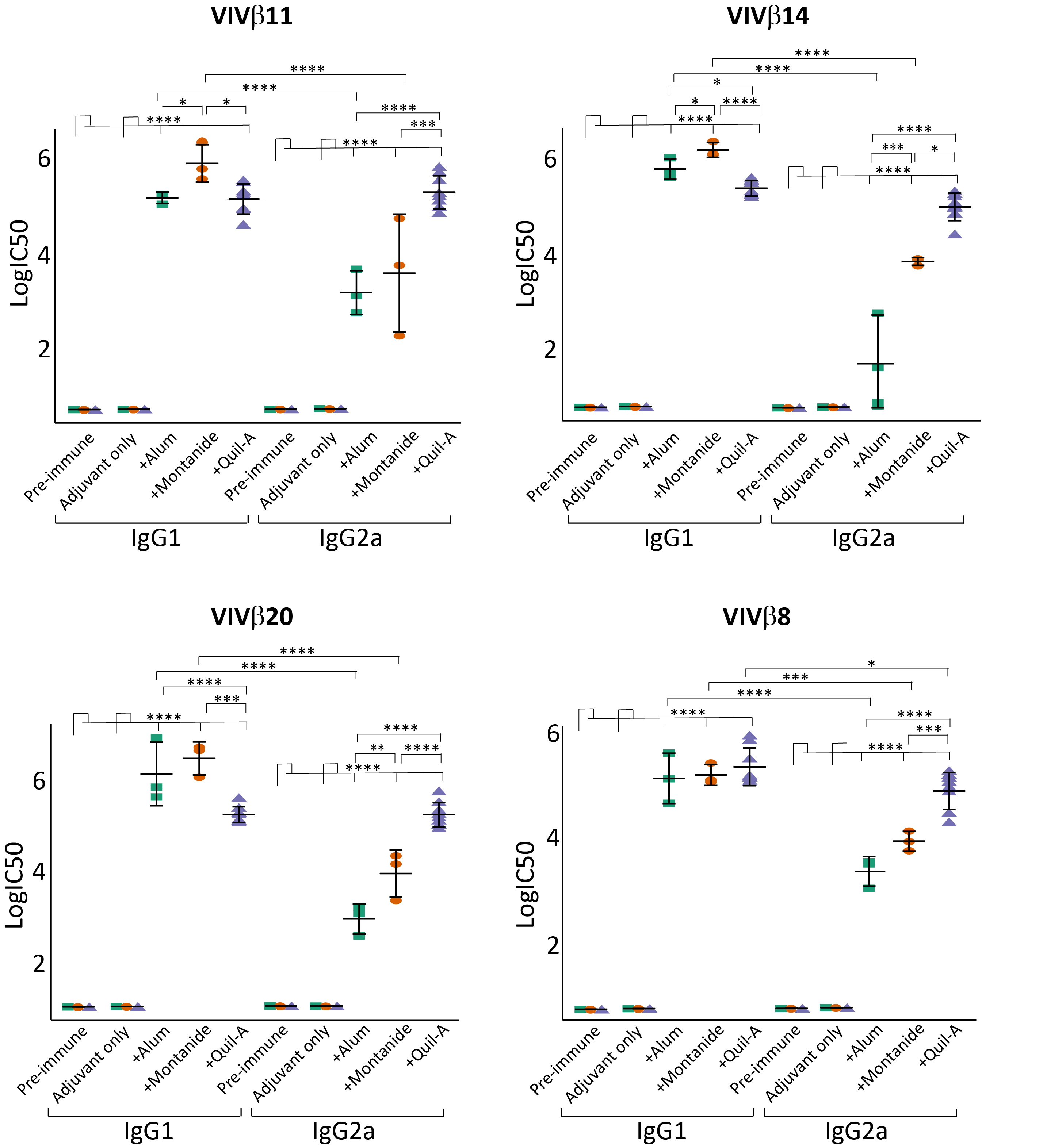
**

**S4 Fig.** **Antibody titres after immunization**. Both IgG1 and IgG2a-specific antibody titres in mice immunized with four different antigens are compared with two negative controls (pre-immune sera and adjuvant-only mice). There is a consistent response for all antigens regardless of the adjuvant used. However, adjuvant choice has a significant effect on antibody titres. Montanide produced significantly higher IgG1 levels than either Alum or Quil-A when applied with VIVβ11*,* VIVβ14 and VIVβ20, but, there was no difference in IgG1 titre between adjuvants when VIVβ8 was used. In contrast, Quil-A produced significantly higher IgG2a titres than either Montanide or Alum when applied with all antigens. Data normality was confirmed with a Shapiro-Wilk test and statistical significance was assessed using a one-tailed ANOVA in R studio. Significance is indicated by asterisks: * (P < 0.05), *** (P < 0.001), **** (P < 0.0001).
