# Supplementary material for: Vivaxin genes encode highly immunogenic, non-variant antigens on the Trypanosoma vivax cell-surface
Source: PLoS Negl Trop Dis. 2022 Sep 21;16(9):e0010791. doi: 10.1371/journal.pntd.0010791 (PMC9529106; doi:10.1371/journal.pntd.0010791)
Supplement: S5 Fig — IgG1 and IgG2a specific antibody titres were measured using two-fold serial dilutions in naturally infected (Cameroon and Kenya) and experimentally infected cattle (Brazil). Antibody levels were also measured in a group of UK cattle, which served as negative controls. IgG1 showed higher levels when compared to IgG2a for both natural and experimental infections with T. vivax. Each graph shows the antibody levels of individual serum, the geometric mean of each group, and the 95% confidence interval. Data normality was confirmed with a Shapiro-Wilk test and statistical significance was assessed using a one-tailed ANOVA in R studio. Significance is indicated by asterisks: **** (P < 0.0001). (DOCX) [file pntd.0010791.s005.docx]

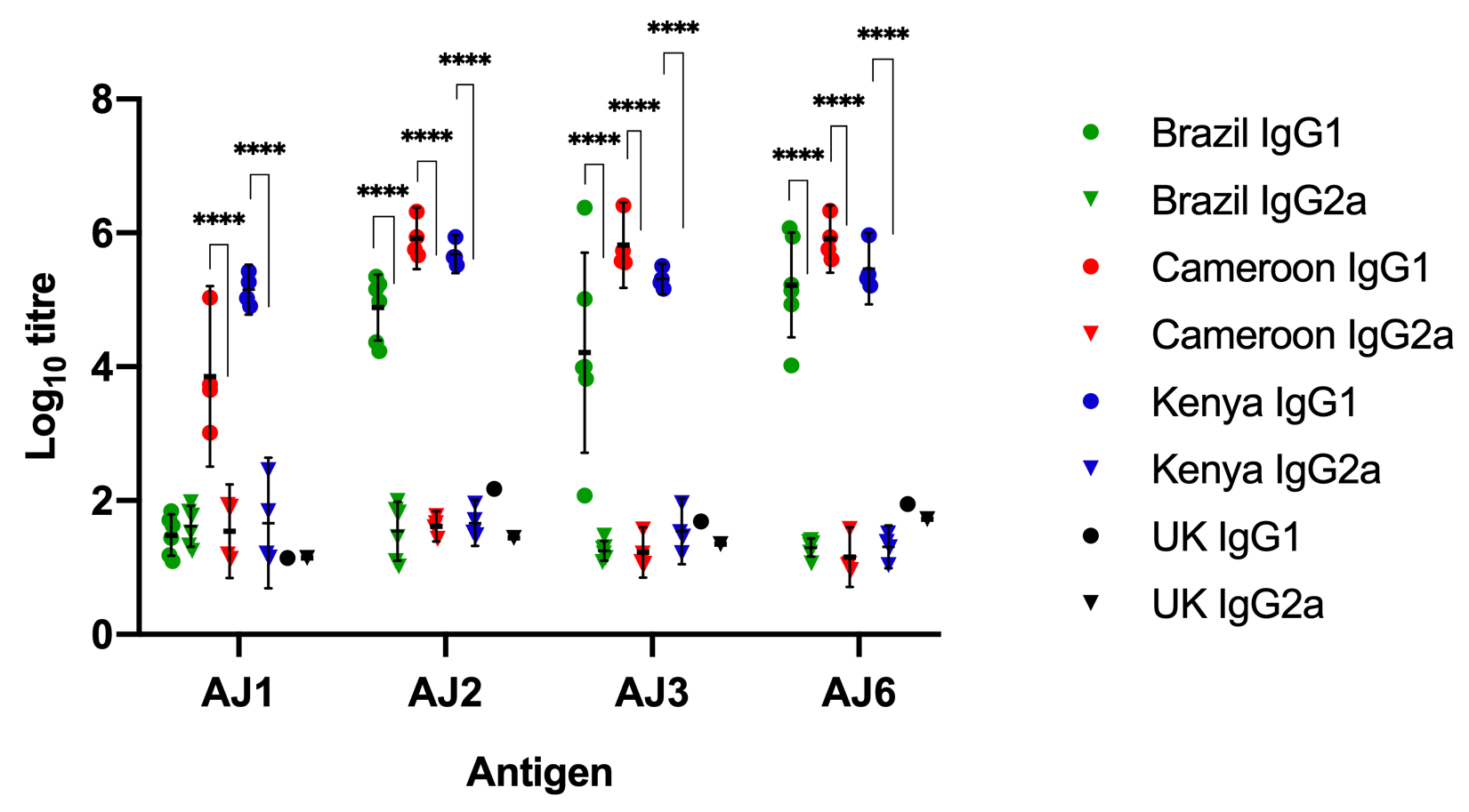


**VIVβ-11**

**VIVβ-14**

**VIVβ-20**

**VIVβ-8**

**1 2 3 4**

Antigen-1

**S5 Fig. Titres of IgG1 and IgG2a isotypes in infected cattle against four antigens, measured by indirect ELISA**. IgG1 and IgG2a specific antibody titres were measured using two-fold serial dilutions in naturally infected (Cameroon and Kenya) and experimentally infected cattle (Brazil). Antibody levels were also measured in a group of UK cattle, which served as negative controls. IgG1 showed higher levels when compared to IgG2a for both natural and experimental infections with *T. vivax*. Each graph shows the antibody levels of individual serum, the geometric mean of each group, and the 95% confidence interval. Data normality was confirmed with a Shapiro-Wilk test and statistical significance was assessed using a one-tailed ANOVA in R studio. Significance is indicated by asterisks: **** (P < 0.0001).
