# Supplementary material for: Vivaxin genes encode highly immunogenic, non-variant antigens on the Trypanosoma vivax cell-surface
Source: PLoS Negl Trop Dis. 2022 Sep 21;16(9):e0010791. doi: 10.1371/journal.pntd.0010791 (PMC9529106; doi:10.1371/journal.pntd.0010791)
Supplement: S6 Fig — Concentrations of four cytokines (TNF-α, IFN-γ, IL-10 and IL-4) were measured in ex vivo mouse splenocyte cultures after stimulation with each vivaxin antigen, co-administrated with one of three adjuvants. Concanavalin A was applied as a positive control. Stimulation with adjuvant only was applied as a negative control. A cross (+) denotes that a value could not be determined. Data normality was confirmed with a Shapiro-Wilk test and statistical significance was assessed using a one-tailed ANOVA in R studio. Significance is indicated by asterisks: ** (P < 0.01), *** (P < 0.001), **** (P < 0.0001). (DOCX) [file pntd.0010791.s006.docx]

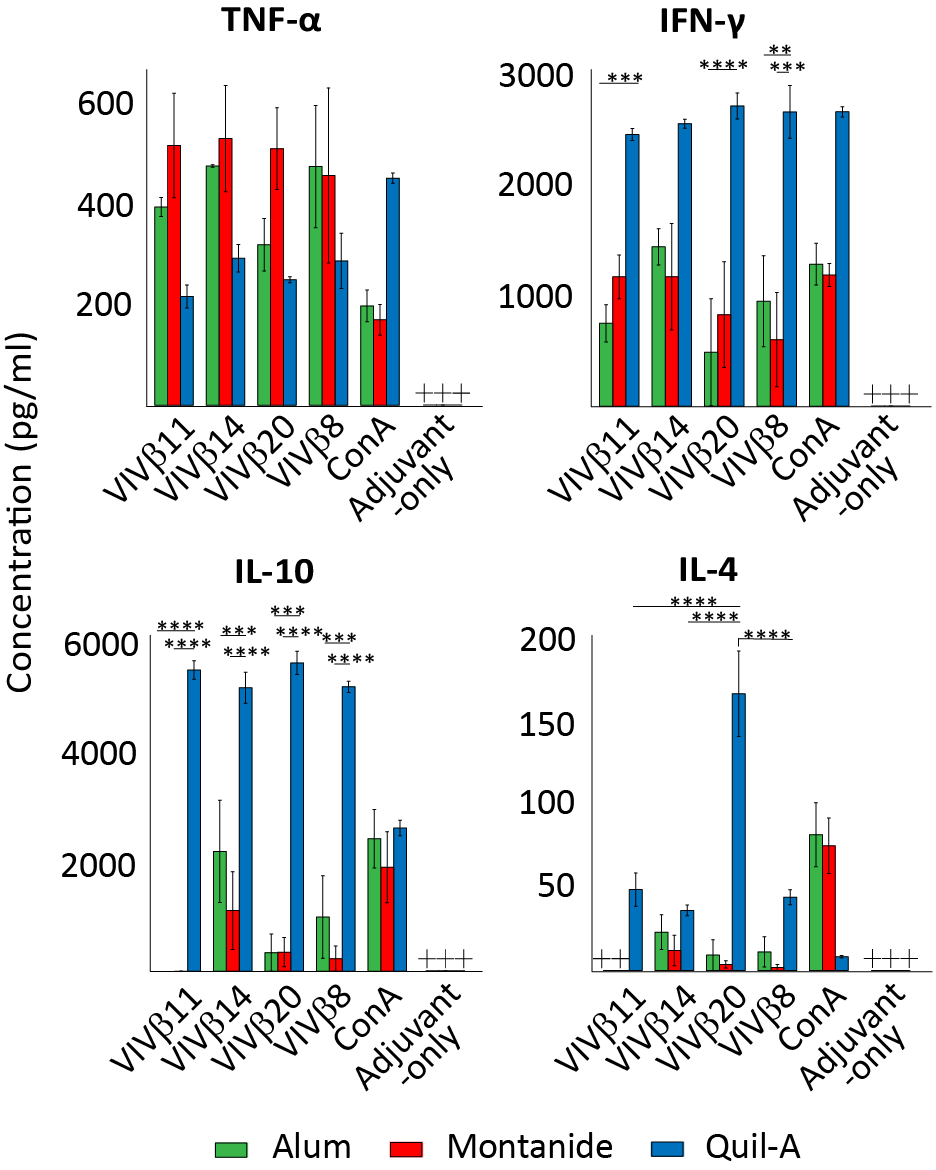


**S6 Fig.** **Cytokine expression after immunization compared for different adjuvants.** Concentrations of four cytokines (TNF-α, IFN-γ, IL-10 and IL-4) were measured in ex vivo mouse splenocyte cultures after stimulation with each vivaxin antigen, co-administrated with one of three adjuvants. Concanavalin A was applied as a positive control. Stimulation with adjuvant only was applied as a negative control. A cross (+) denotes that a value could not be determined. Data normality was confirmed with a Shapiro-Wilk test and statistical significance was assessed using a one-tailed ANOVA in R studio. Significance is indicated by asterisks: ** (P < 0.01), *** (P < 0.001), **** (P < 0.0001).
