# Supplementary material for: Vivaxin genes encode highly immunogenic, non-variant antigens on the Trypanosoma vivax cell-surface
Source: PLoS Negl Trop Dis. 2022 Sep 21;16(9):e0010791. doi: 10.1371/journal.pntd.0010791 (PMC9529106; doi:10.1371/journal.pntd.0010791)
Supplement: S7 Fig — A. Luciferase intensity from VIVβ8+Quil-A-vaccinated animals was significantly lower than the adjuvant-only control group at 6 dpi (p = 0.016), with means of 1.32x10^8^ and 1.71x10^8^ p/s respectively. On subsequent days, there were no significant differences between luminescence values of vaccinated and control groups. B. Kaplan-Meir survival curve (%) of both groups during the course of infection. C. Bioluminescence values from VIVβ8-vaccinated and control animals compared. D. Isotype IgG profiling in challenged animals culled at 8 and 9 dpi. E. Cytokine levels in challenged animals culled at 8 and 9 dpi. There were no significant changes in TNF-α, IFN-γ and IL-10 concentrations between 8 dpi and 9 dpi. There was a significant rise in IL-4 concentration, with undetectable values at 8 dpi and an average concentration of 7.83pg/ml at 9 dpi (p = 0.028). The comparison between 8 dpi and 9 dpi also showed pronounced changes in IL-10 and IL-4 levels in the control group stimulated with ConA (p = 3.40E-04 and p = 1.78E-04 for IL-10 and IL-4, respectively). In all cases, cytokine concentration from splenocytes stimulated with VIVβ8 was lower than the control group stimulated with ConA, except for IL-4 levels at 9 dpi. Data normality was confirmed with a Shapiro-Wilk test and statistical significance was assessed using a one-tailed ANOVA (panel D) or paired t-test (panel E) in R studio. Significance is indicated by asterisks: * (P < 0.05), ** (P < 0.01), *** (P < 0.001), **** (P < 0.0001). (DOCX) [file pntd.0010791.s007.docx]

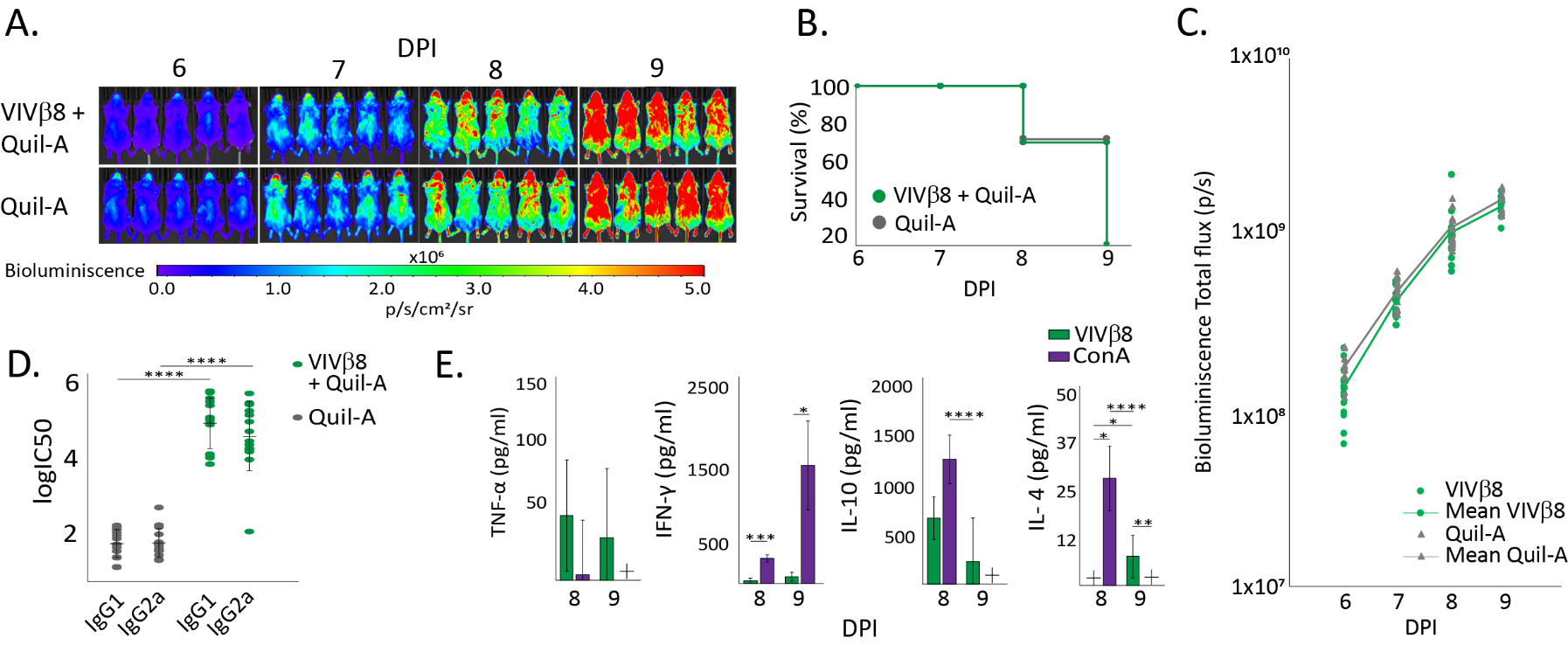


**S7 Fig.** **VIVβ8 vaccination and challenge experiment in BALB/c mice repeated with a larger cohort (n=15/group) and antigen dose (50µg); protocol as described in methods**. **A.** Luciferase intensity from VIVβ8+Quil-A-vaccinated animals was significantly lower than the adjuvant-only control group at 6 dpi (p =0.016), with means of 1.32x108 and 1.71x108 p/s respectively. On subsequent days, there were no significant differences between luminescence values of vaccinated and control groups. **B.** Kaplan-Meir survival curve (%) of both groups during the course of infection. **C.** Bioluminescence values from VIVβ8-vaccinated and control animals compared. **D.** Isotype IgG profiling in challenged animals culled at 8 and 9 dpi. **E.** Cytokine levels in challenged animals culled at 8 and 9 dpi. There were no significant changes in TNF-α, IFN-γ and IL-10 concentrations between 8 dpi and 9 dpi. There was a significant rise in IL-4 concentration, with undetectable values at 8 dpi and an average concentration of 7.83pg/ml at 9 dpi (p =0.028). The comparison between 8 dpi and 9 dpi also showed pronounced changes in IL-10 and IL-4 levels in the control group stimulated with ConA (p =3.40E-04 and p =1.78E-04 for IL-10 and IL-4, respectively). In all cases, cytokine concentration from splenocytes stimulated with VIVβ8 was lower than the control group stimulated with ConA, except for IL-4 levels at 9 dpi. Data normality was confirmed with a Shapiro-Wilk test and statistical significance was assessed using a one-tailed ANOVA (panel D) or paired t-test (panel E) in R studio. Significance is indicated by asterisks: * (P < 0.05), ** (P < 0.01), *** (P < 0.001), **** (P < 0.0001).
