# Supplementary material for: Vivaxin genes encode highly immunogenic, non-variant antigens on the Trypanosoma vivax cell-surface
Source: PLoS Negl Trop Dis. 2022 Sep 21;16(9):e0010791. doi: 10.1371/journal.pntd.0010791 (PMC9529106; doi:10.1371/journal.pntd.0010791)
Supplement: S8 Fig — (A) Localization of VIVβ8 and the unspecific surface counterstain mCLING in red blood cells (RBC) from T. vivax-infected mice. Representative images of RBC stained with either pre-immune or post-immune rabbit polyclonal antisera. Middle row shows the major localization pattern of VIVβ8 in RBC; protein accumulates in the central concave surface. Bottom row shows an example of leaking RBC. Differential increased contrast (DIC); DAPI DNA counterstain; VIVβ8 (secondary antibody AF555-conjugated) and merged channels. Scale bars; 5 μm. (B) 3D z-stack reconstructions of mouse erythrocyte cells and corresponding orthogonal (X-Z and X-Y) views from the stacks. Orthogonal views reflect the accumulation of VIVβ8 signal at the inner concave cell membrane. Scale bars; 5 μm. (DOCX) [file pntd.0010791.s008.docx]

**
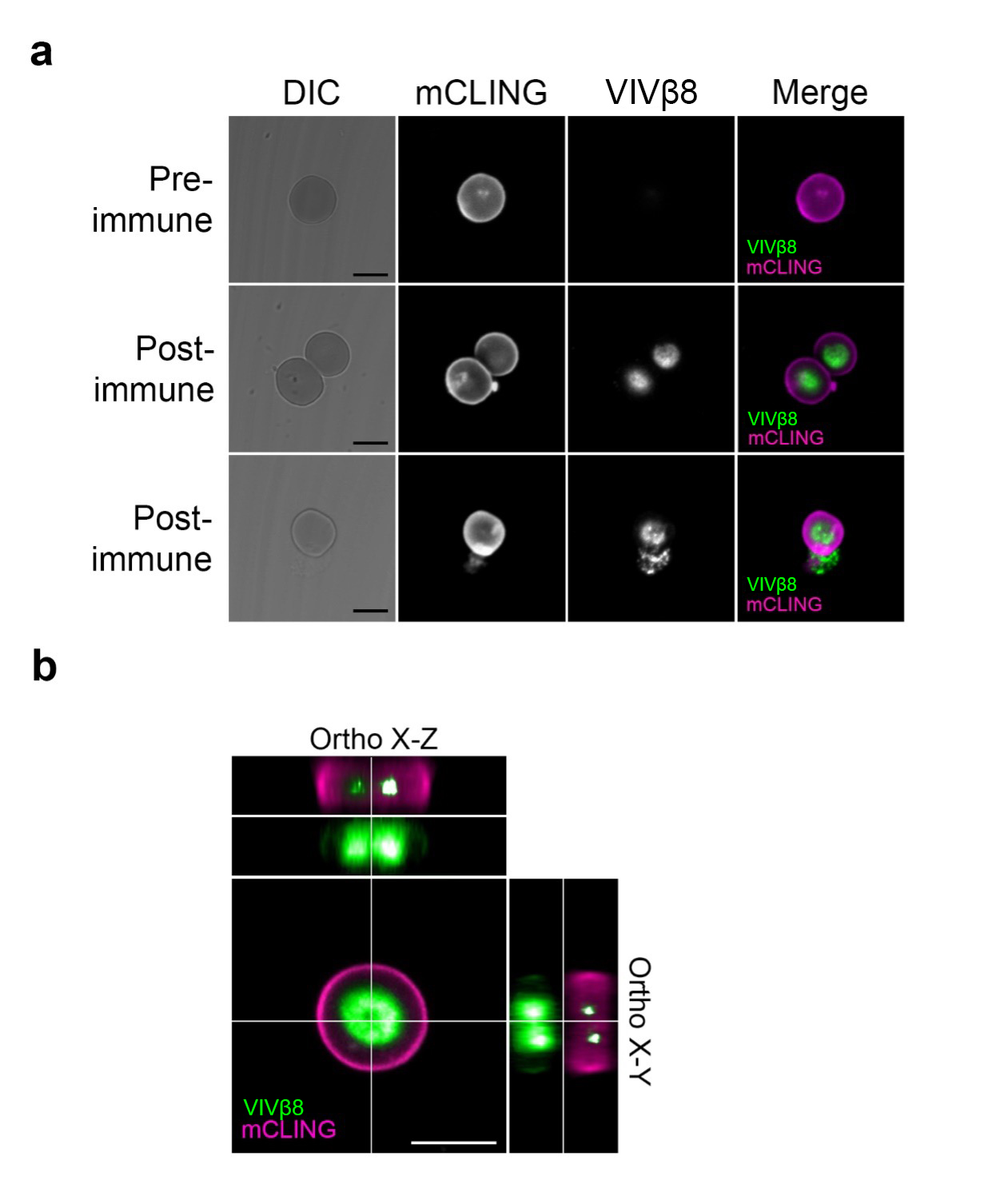
**

**S8 Fig. Cellular localization of VIVβ8** **antigen on the surface of murine erythrocytes**. **(A)** Localization of VIVβ8 and the unspecific surface counterstain mCLING in red blood cells (RBC) from *T. vivax*-infected mice. Representative images of RBC stained with either pre-immune or post-immune rabbit polyclonal antisera. Middle row shows the major localization pattern of VIVβ8 in RBC; protein accumulates in the central concave surface. Bottom row shows an example of leaking RBC. Differential increased contrast (DIC); DAPI DNA counterstain; VIVβ8 (secondary antibody AF555-conjugated) and merged channels. Scale bars; 5 μm. **(B)** 3D z-stack reconstructions of mouse erythrocyte cells and corresponding orthogonal (X-Z and X-Y) views from the stacks. Orthogonal views note the VIVβ*8* signal originates in the inner concave cytoplasm. Scale bars; 5 μm.
